# Initial Development of a Multidimensional Computerized Adaptive Test for Intensive Longitudinal Assessment of Suicide Risk: Development and Usability Study

**DOI:** 10.2196/76544

**Published:** 2025-11-26

**Authors:** Kenneth McClure, Brooke A Ammerman, Cheng Liu, Miguel Blacutt, Connor O'Brien, Ross Jacobucci

**Affiliations:** 1 Department of Mathematics and Statistics, University of Wyoming, 1000 E University Ave, Laramie, WY, 82071, United States, 1 3077666831; 2 Department of Psychology, University of Wisconsin–Madison, Madison, WI, United States; 3 Department of Psychology, University of Notre Dame, South Bend, IN, United States; 4 Center for Healthy Minds, University of Wisconsin–Madison, Madison, WI, United States

**Keywords:** ecological momentary assessment, computerized adaptive testing, suicide, measurement, participant burden

## Abstract

**Background:**

Intensive longitudinal designs support temporally granular study of processes, making methods like ecological momentary assessment (EMA) increasingly common in medical and behavioral science. However, the repetitive and intensive measurement strategies associated with these designs increase participant burden, which limits the breadth and precision of EMA surveys. This is particularly problematic for complex clinical phenomena, such as suicide risk, which research has shown is multidimensional and fluctuates over narrow time intervals (eg, hours). To overcome this limitation, we proposed the Computerized Adaptive Test for Suicide Risk Pathways (CAT-SRP), which supports the simultaneous assessment of multiple empirically informed risk domains and facilitates personalized survey content.

**Objective:**

The objective of this study is to develop, calibrate, and pilot the first multidimensional computerized adaptive test for suicidal thoughts and related psychosocial risk factors in intensive longitudinal designs like EMA.

**Methods:**

A web-based assessment platform was developed to adaptively administer the CAT-SRP. CAT-SRP items were modified from existing validated instruments to support administration in intensive longitudinal designs. The item bank was developed in line with major ideation-to-action theories of suicide and consultation with experts outside the study team. Exploratory item factor analysis was used to identify dimensionality of the item bank. Item parameters were calibrated using a multidimensional graded response model in a large cross-sectional community sample (n=1759, 36.33% with a history of suicidal thoughts). Following calibration, the CAT-SRP was evaluated in an EMA study of participants with a past month history of suicidal thoughts (n=29 across 2134 observations). Adaptive testing used D-optimal item selection, a dual variable-length stopping criterion, and Maximum a Posteriori (MAP) scoring. Descriptive statistics and mixed effects models were used to examine CAT-SRP performance (eg, efficiency and survey overlap) and relationships among CAT-SRP domain scores.

**Results:**

The calibration study identified 2 suicidal thought domains (active and passive thoughts) and 12 risk factor domains: humiliation, loneliness, anger, pain, defeat, impulsivity (ie, negative urgency), entrapment, distress tolerance, perceived burdensomeness, thwarted belongingness, aggression, and a positively valenced method factor. Domain information was the highest between average to high levels of domain scores. Study 2 showed that the CAT-SRP (1) administered surveys with low to moderate item overlap, (2) incurred low participant burden, and (3) may improve near-term prediction of suicidal thoughts relative to traditional EMA measurement. Most EMA surveys reached the maximum length, 50 questions, highlighting a need to refine selection and stopping rules.

**Conclusions:**

The CAT-SRP effectively personalized EMA survey content to respondents, which reduces the repetitiveness and perceived burden of intensive longitudinal research designs. Continuous domain scores from multidimensional computerized adaptive testing (MCAT) also provided more nuanced measurement compared to traditional approaches that struggle with zero-inflation in EMA and appeared to produce stronger predictive relationships. Overall, the CAT-SRP demonstrated strong methodological advantages to use CAT for intensive longitudinal data collection.

## Introduction

### Background

Attempts to understand suicide, a growing public health concern [1], have led to the development of several theoretical models outlining factors contributing to suicidal thoughts and behaviors. Although theoretical models, such as the Interpersonal Theory of Suicide (ITS) [2], the Three-Step Theory of Suicide (3ST) [3], and the Integrated Motivational-Volitional (IMV) [4], have received notable empirical attention [5], the translation and application to real-time risk processes remains limited. Research strongly supports that suicidal thinking is dynamic [6], with transitions between suicidal thinking and behavior potentially occurring within hours [7]. However, a comprehensive, temporally dynamic examination of these theoretical models is lacking, limiting both suicide risk assessment accuracy and advances toward near-term risk prediction.

The increasing accessibility of intensive time sampling methodologies (eg, ecological momentary assessment [EMA]) has begun to bridge this gap by capturing the fluctuating nature of suicide risk in real-time. However, limitations common in EMA protocols have hindered a comprehensive assessment of risk processes outlined in theoretical models. EMA studies typically rely on a limited, repetitive subset of predictors, frequently assessed using short-form or single-item measures that lack thorough validation [8,9]. This approach compromises measurement precision [10,11], decreases statistical power [12-14], biases parameter estimates (eg, attenuation) [12,13], and masks potential nonlinear effects [12-14]. Further, the repetitive nature of item content may increase participant burden, leading to decreased compliance rates and diminished data quality. Reliance on a narrow set of items also constrains the evaluation and refinement of theoretical models, limiting the ability to identify subgroups with distinct risk trajectories or explore alternative pathways that may not apply uniformly across populations. This restriction further hinders the development of personalized models that could enhance prediction accuracy and intervention strategies. These limitations undermine confidence in findings and hinder the interpretability of results, ultimately reducing their impact on suicide risk assessment and intervention.

Computerized adaptive testing (CAT) [15] offers a systematic and efficient approach to momentary risk assessment that addresses such limitations [16]. CAT uses formal psychometric models, typically item response models [17], to dynamically administer subsets of items from larger precalibrated item banks, with the objective of maximizing information (ie, precision) of underlying latent risk variables. This results in more efficient and personalized measurement [18]. Although prior CATs for suicide exist [19-21], each was developed as a suicide risk screening for a single time point (ie, primary care or emergency department visit), neither to comprehensively evaluate risk factors nor to be used repeatedly for momentary assessment. For example, the Computerized Adaptive Test-Suicide Scale (CAT-SS) [21] was developed as a brief screening instrument to identify individuals with nonnegligible levels of suicide risk in health care settings. The CAT-SS item pool consists predominantly of depression and anxiety items, with a small set of suicide-specific items, and uses a bifactor measurement model to assess a general risk factor that is shared among the depression, anxiety, and suicide items; other measures take a similar approach [19,20]. Although these instruments are effective at assessing overall suicide risk, they do not provide scores for individual risk factors, which are critical for understanding the development and dynamics of suicidal thoughts and behaviors. Recent work has implemented CAT in assessing symptom severity in patient-reported outcomes research using an EMA design [22], highlighting the potential for application in suicide research.

To leverage the advantages of CAT for studying suicide risk factors in intensive longitudinal designs, we developed the Computerized Adaptive Test of Suicide Risk Pathways (CAT-SRP). The CAT-SRP is the first CAT designed for monitoring the fluid nature of suicide risk factors in intensive time sampling approaches, like EMA. Unlike existing suicide CATs [20,21] which focus on screening overall risk for suicide at a single time point (eg, primary care or emergency department visit) using bifactor measurement models, the CAT-SRP emphasizes the assessment of individual risk factors (eg, hopelessness and loneliness) in intensive longitudinal designs using a multidimensional graded response model (MGRM) [23] with correlated risk factors. Specifically, the CAT-SRP is intended to assess risk factors proposed in 3 predominant ideation-to-action models of suicide [2-4] and other well-established risk factors for near-term suicidal thoughts and behaviors (STBs; eg, anger and stress [8,9,24]), as well as suicidal thoughts. The CAT-SRP is not predominantly grounded in any of the ITS, 3ST, or IMV, but instead aims to provide an instrument for assessing and comparing risk pathways from each of the theoretical models. While prior work has highlighted the salience of these factors in predicting suicide risk, no EMA study has directly and comprehensively examined the confluence of these risk factors within a single study protocol.

Using CAT in suicide EMA research also introduces key ethical considerations. First, variable-length CAT methods, which are typically more efficient [18], may result in longer surveys for some respondents. The number of items needed to terminate measurement depends on both the underlying levels of the latent variable for a given respondent and the characteristics of the item bank. Surveys will tend to be shorter when the item bank contains items that provide high information at a participant’s risk level. Thus, to limit participant burden, it is often advantageous to use a dual stopping rule [25] and specify a maximum survey length. Additionally, for research contexts where participant risk is monitored and study staff may need to intervene, such as in suicide EMA research [26,27], additional care in adaptive EMA survey design is needed. A fully adaptive approach may exclude items used in risk monitoring. Thus, until adaptive risk monitoring approaches have been thoroughly validated, a static validated assessment of acute risk (eg, imminent self-harm or death) should be used across assessments like in traditional EMA research.

### Study Aims

The present paper aims to develop and validate the CAT-SRP through 2 complementary studies. Study 1 develops and calibrates the CAT-SRP item bank using a large community sample. Study 2 pilots the feasibility, acceptability, and utility of the CAT-SRP as a measure of momentary suicidal ideation and associated risk factors in an EMA design using a sample of individuals with a past month history of STBs. By developing this comprehensive adaptive measurement system, we aim to advance both the methodological rigor of suicide risk assessment in intensive longitudinal research and our theoretical understanding of how risk factors dynamically relate to suicidal thoughts and behaviors.

## Methods

### Study 1

#### Participants

Participants (n=2498) were recruited using Prime Panels (CloudResearch) [28]. Participants were required to be at least 18 years of age, speak English, and reside in the United States. After providing informed consent, participants were asked to complete the full CAT-SRP item pool and demographic items. Respondents failing automated bot detection (ie, reCAPTCHA; n=14), providing completely missing responses (n=38), completing the entire battery in under 7 minutes (n=103), or failing more than 1 out of 6 attention checks (n=720) were excluded from analyses (note some respondents were flagged in multiple checks). This resulted in a final sample of n=1759 adults (mean age 46, SD 16.9 years). See Table 1 for sample demographics. Of the final sample, 36.33% of respondents reported a lifetime history of suicidal ideation, planning, or attempts. Missing data for CAT-SRP item responses were minimal (0.85%).

**Table 1.** Study demographics.

Variable	Study 1 (n=1759)		Study 2 (n=29)	
Gender, n (%)				
Woman	957 (54.41)		18 (62.07)	
Man	787 (44.74)		10 (34.48)	
Transgender	12 (0.68)		0 (0)	
Prefer not to Answer	3 (0.17)		0^a^ (0)	
Ethnicity, n (%)				
Non-Hispanic or Latinx	1314 (74.7)		3 (10.34)	
Hispanic or Latinx	427 (24.28)		26 (89.66)	
Prefer not to answer	18 (1.02)		0 (0)	
Race				
White	1149 (65.32)		20 (68.97)	
Black or African American	439 (24.96)		6 (20.69)	
Multiracial	62 (3.52)		2 (6.70)	
Asian	22 (1.25)		0 (0)	
American Indian or Alaskan Native	17 (0.97)		0 (0)	
Native Hawaiian or Pacific Islander	3 (0.17)		0 (0)	
Not listed or prefer not to answer	67 (3.81)		0 (0)	

a One participant identified as nonbinary.

#### Procedure

Target domains for the CAT-SRP were selected based on (1) predominant ideation-to-action theories of suicide [2-4] and (2) consultation with 4 experts in suicide research. Specifically, psychosocial risk factors central to the ITS, 3ST, or IMV, such as hopelessness, thwarted belongingness, defeat, and entrapment, were chosen as target domains. Additional risks identified by experts as potentially important for studying suicide risk in EMA (ie, anger, aggression, negative urgency, and perceived stress) were also included. In total, 12 preliminary risk factor domains were identified. CAT-SRP items (J=200) for these domains were adapted from existing measures of corresponding constructs (see the Measures section). In addition to the 12 STB risk domains, the CAT-SRP also includes passive and active suicidal ideation domains (J=18). Measurement models for the risk factors and suicidal thoughts were calibrated separately to prevent interpretational confounding [29].

To support delivery in future intensive longitudinal designs, all CAT-SRP items were modified to conform to a 5-point Likert scale and include a day-level temporal referent (ie, “Today, …”). Maintaining a consistent response scale may also reduce participant burden in intensive longitudinal designs [30]. Original item phrasing and modified phrasing for the CAT-SRP are available on OSF [31]. Unlike previous high-dimensional adaptive tests for suicide risk, which focus on assessing general levels of risk [20,21], the CAT-SRP prioritizes assessing individual risk domains, as well as suicidal thoughts. Thus, domains were conceptualized as correlated factors rather than specific factors in a bifactor structure. The final CAT-SRP item pool and calibrated item parameters can also be found on OSF.

#### Measures

##### Perceived Burdensomeness

Eight perceived burdensomeness (PB) items were included in the initial CAT-SRP item pool. Seven items were adapted from the Interpersonal Needs Questionnaire (INQ) [32] and one item was adapted from an ultra-brief measure of suicide risk [33].

##### Thwarted Belongingness

Fifteen items assessing thwarted belongingness (TB) were included in the initial CAT-SRP item pool. Eight items were adapted from the INQ [32] and 7 items were adapted from the Thwarted Belongingness Scale [34].

##### Loneliness

Twenty items assessing loneliness were included in the initial CAT-SRP item pool and adapted from the UCLA (University of California, Los Angeles) Loneliness Scale [35].

##### Hopelessness

Twenty items assessing hopelessness were included in the initial CAT-SRP item pool. Ten items were adapted from the state subscale of the State-Trait Hopelessness Scale [36]. Ten items were adapted from the Brief Hopelessness Scale [37]; 5 of these items were adapted from the positive subscale (ie, hope) and 5 from the negative subscale.

##### Defeat and Entrapment

Sixteen items assessing defeat and 16 items assessing entrapment were included in the initial CAT-SRP item pool and were adapted from the Defeat and Entrapment Scales, respectively [38].

##### Humiliation

Thirty-two items assessing humiliation were included in the initial CAT-SRP item pool and were modified from the Humiliation Inventory [39].

##### Psychological Pain

Thirteen items assessing psychological pain were included in the initial CAT-SRP item pool and were adapted from the Psychache Scale [40].

##### Anger and Aggression

Twenty items assessing anger and four items assessing aggression were included in the initial CAT-SRP item pool and were adapted from the Patient-Reported Outcomes Measurement Information System (PROMIS) anger scale [41].

##### Perceived Stress

Ten items assessing perceived stress were included in the initial CAT-SRP item pool and were adapted from the Perceived Stress Scale [42].

##### Negative Urgency

Twelve items assessing negative urgency were included in the initial CAT-SRP item pool and were adapted from the negative urgency subscale of the Urgency, (Lack of) Premeditation, (Lack of) Perseverance, Sensation Seeking, and Positive Urgency (UPPS-P) Impulsive Behavior Scale [43].

##### Distress Tolerance

Fifteen items assessing distress tolerance were included in the initial CAT-SRP item pool and were adapted from the Distress Tolerance Scale [44].

##### Suicidal Ideation

Eighteen items assessing passive and active suicidal ideation were included in the initial CAT-SRP item pool. Nine items assessing passive ideation and nine items assessing active ideation were adapted from the passive and active suicidal ideation scale (PASIS) subscales, respectively [45].

### Analytic Plan

Parallel analysis and scree plots using polychoric correlation matrices were used to explore CAT-SRP dimensionality. Polychoric correlation matrices with pairwise complete data were obtained using the polycor package [46]. The psych package [47] was used for parallel analyses. Exploratory item factor analysis (EIFA) was conducted for plausible model dimensionalities as suggested by parallel analysis. Interpretability of factor loading patterns, alongside parallel analysis, was used to guide selection among models with similar performance in EIFA.

After identifying item bank dimensionality, a confirmatory MGRM was fit using the mirt package [48] with Metropolis-Hastings Robbins-Monro estimation to calibrate item parameters [49,50]. Items with loadings of >.20 in the EIFA were specified to load onto corresponding domains in the MGRM, allowing within-item multidimensionality; otherwise, cross-loadings were constrained to 0. Passive and active STB items were calibrated using a 2-dimensional MGRM. Item difficulty and discrimination parameters were extracted to facilitate adaptive testing. Study data, including the CAT-SRP item bank and the original CAT-SRP blueprint, is available on OSF [31]. This study was not preregistered.

### Ethical Considerations

All study procedures were reviewed and deemed exempt by the University of Notre Dame Institutional Review Board (protocol number 22-09-7402). A waiver of informed consent was obtained to preserve participant anonymity. Prior to study tasks, participants were informed of study details. Participants were informed that they were free to discontinue participation at any time by closing the survey window. Participants were compensated US $3.25.

### Study 2

#### Participants

Participants (n*=*29) were recruited from online sources, including Facebook (Meta Platforms, Inc), Instagram (Meta Platforms, Inc), Reddit (Reddit, Inc), and Craigslist (Craigslist, Inc) to participate in a study on moods, feelings, and thoughts in daily life. Prospective participants completed a brief semistructured phone interview with study staff to determine eligibility. Inclusion criteria included (1) being 18 years or older, (2) having a past-month history of suicidal thoughts (eg, “Have you had thoughts of killing yourself in the past month?”), (3) speaking English, (4) being located in the United States, and (5) owning a smartphone. Participants were adults in the United States (mean age 34.62, SD 10.13 years). See Table 1 for gender, ethnicity, and race sample composition. Overall, 22 out of 29 (75.86%) of participants self-reported having received a mental health diagnosis, and 10 out of 29 (34.48%) participants reported prior hospitalization for psychiatric reasons.

#### Procedure

Participants attended one virtual baseline session before the EMA period. Following informed consent, participants completed a battery of self-report measures and the full CAT-SRP item bank during the initial virtual session. Participants also created an account on the CAT-SRP website to allow for EMA data collection. Responses to CAT-SRP items at baseline were used to initialize the CAT algorithm for EMA surveys (see CAT Algorithm section). The EMA period began the morning following the virtual session. Participants were sent 6 EMA survey links per day for 21 days via an automated SMS messaging system. Survey timing was randomized within six 2-hour intervals between 9 AM and 9 PM; adjacent surveys were required to occur at least 15 minutes apart. Participants received one reminder 15 minutes after the initial prompt if they had not completed the survey. All EMA notifications were delivered via text message and included a link to the CAT-SRP web platform where participants completed the adaptive CAT-SRP survey and risk monitoring items (see “Momentary Suicide Risk” measures below). See Figure S1 in Multimedia Appendix 1 for a flowchart of the design of study 2.

#### Risk Monitoring

If responses suggesting high suicide risk were detected during the EMA period (ie, suicidal intent reported as >8 out of 10, a reported plan to attempt suicide that day, or a reported suicide attempt), this triggered an email alert to the research team (resulting in n=4 alerts to the study team). A trained member of the study team then followed up via phone to conduct a comprehensive risk assessment; acute suicide risk was categorized based on established suicide risk management protocols [51]. As appropriate, potential call outcomes included resource review, development of a safety plan, or transportation for an in-person safety assessment (eg, emergency department). All participants were provided resources at the onset of the study. Participants reporting high-risk responses were also provided with a comprehensive resource list when high risk was detected.

#### Follow-Up and Compensation

Following the EMA data collection, participants were sent an online survey to assess response burden. Participants were compensated US $20 for completing the virtual assessment, US $150 for completing the 21-day EMA period (with a US $15 bonus for completing at least 75% of surveys), and US $10 for completing the final online survey. Thus, participants were compensated between US $170-US $195 in total.

#### CAT Algorithm

The CAT-SRP items were administered adaptively during EMA surveys. Baseline estimates of latent severity (ie, $\hat{\theta }$) were used to initialize the CAT-SRP at the first EMA survey; all follow-up surveys were initialized using the final estimates of the preceding measurement occasion. Given the multidimensional nature of the item bank, items were selected sequentially to maximize the determinant of the Fisher Information matrix (ie, D-optimal item selection). This approach favors items that provide information on multiple latent variables [52] and was found to better recover $\hat{\theta }$ in high-dimensional measures with shorter tests [53]. To ensure that all CAT-SRP domains were assessed directly at every measurement occasion, a “warm-up” period was implemented where the most informative item (ie, D-optimal) from each domain was administered. Thus, at least one item from each of the 14 domains was administered on every occasion. Following this warm-up period, items were adaptively administered until a stopping criterion was reached.

The CAT-SRP was administered as a variable-length CAT with additional constraints. A dual stopping criterion, which (1) evaluated observed measurement precision (ie, D-rule) and (2) convergence of $\hat{\theta }$ across all domains for 2 sequential item administrations (ie, CT-rule), was used to terminate the test [25]. More formally, the D-rule is given by

$n=inf_{I}\;\left\{j\;\geq 1:\;det\left[\sum_{j=1}^{n}I_{j}\left(y_{j}|{\hat{\theta }}^{n}\right)\right]\geq \;{\left(\frac{v}{c}\right)}^{2}\right\}$(1)

where

$v=\;\frac{2\pi^{\frac{m}{2}}{\left[\chi_{m}^{2}\left(\alpha \right)\right]}^{\frac{m}{2}}}{m\Gamma \left(\frac{m}{2}\right)}$(2)

and *c* represents the maximum allowable confidence ellipsoid volume (typically an *m*-dimensional unit ball), and α is the desired significance level [25]. In this study, *m*=14 and *α*=.05. For the CT rule, the survey ended if the changes to all $\hat{\theta }$ were less than 0.01 for 2 consecutive item administrations. A maximum test length of 50 items was enforced if neither the D-rule nor CT rule was satisfied. Thus, CAT-SRP survey lengths ranged from 14‐50 items.

#### Baseline Measures: CAT-SRP

During the baseline virtual session, participants completed all 207 items retained from the initial CAT-SRP item pool (see study 1 for more details).

#### EMA Measures

##### EMA CAT-SRP

CAT-SRP items were adaptively administered during EMA surveys. Temporal referents for EMA CAT-SRP items were modified to be “Since the last prompt…” “Since the last prompt” was chosen because it was considered to be more consistent with the temporal referent in the calibration sample (“Today…”) than other alternatives (eg, “right now” and “currently”) while still appropriate for EMA designs. The CAT-SRP uses a variable-length sequentially adaptive item selection algorithm (see CAT Algorithm section). Thus, survey length and content potentially differed for each respondent and survey occasion. At each measurement occasion, however, at least one item from each of the CAT-SRP domains (ie, risk and suicidal ideation) was administered, resulting in a minimum adaptive survey length of 14 items. A maximum number of 50 items was also specified.

##### Momentary Suicide Risk

In addition to the adaptive CAT-SRP suicidal ideation items, 3 items assessing momentary STB risk were administered at each prompt: “Do you intend to act on your suicidal thoughts? (1-10),” “Do you plan to attempt suicide today or tonight? (yes or no),” “Since the last prompt have you attempted suicide (yes or no)?” These items were not evaluated as part of the CAT-SRP but used only for the purpose of identifying imminent suicide risk.

### End-of-Study Measure: Participant Subjective Burden

Subjective burden was assessed using 9 questions in the end-of-study survey (see Table 2). Additional questions assessing reaction to research participation were also asked as part of a larger questionnaire [54] but not analyzed in this study.

**Table 2.** Descriptive statistics for subjective burden item responses.

Survey Item	1, n (%)	2, n (%)	3, n (%)	4, n (%)	5, n (%)	Mean (SD)	Median (IQR)
Did you feel overwhelmed by the number of survey questions^a^?	10 (34.5)	9 (31.0)	8 (27.6)	2 (6.9)	0 (0)	2.07 (0.96)	2 (1-3)
Did you feel annoyed or frustrated by the number of survey questions^a^?	10 (34.5)	12 (41.4)	5 (17.2)	2 (6.9)	0 (0)	1.97 (0.91)	2 (1-2)
Did you feel annoyed or frustrated by the number of surveys^a^?	15 (53.6)	4 (14.3)	8 (28.6)	1 (3.6)	0 (0)	1.82 (0.98)	1 (1-3)
How often did you skip a survey because it felt like too many surveys^b^?	15 (51.7)	6 (20.7)	7 (24.1)	1 (3.4)	0 (0)	1.79 (0.94)	1 (1-3)
How often did you skip a survey because it felt like too many survey questions?^b^	19 (67.9)	7 (25)	2 (7.1%)	0 (0)	0 (0)	1.39 (0.63)	1 (1-2)
I found participating boring.^c^	8 (27.6)	8 (27.6)	7 (24.1)	6 (20.7)	0 (0)	2.38 (1.12)	2 (1-3)
The study procedures took too long^c^	9 (31)	9 (31)	10 (34.5)	1 (3.4)	0 (0)	2.10 (0.90)	2 (1-3)
Participating in the study was inconvenient to me^c^	9 (31)	13 (44.8)	6 (20.7)	1 (3.4)	0 (0)	1.97 (0.82)	2 (1-2)
Had I known in advance what participating would be like, I still would have agreed to participate^c^	0 (0)	0 (0)	3 (10.3)	7 (24.1)	19 (65.5)	4.55 (0.69)	5 (4-5)

a 1=very slightly/not at all, 2=a little, 3=moderately, 4=quite a bit, 5=extremely.

b 1=not at all, 2=less than once per week, 3=multiple times per week, 4=once per day, 5=multiple times per day.

c 1=strongly disagree, 2=disagree, 3=neutral, 4=agree, 5=strongly agree.

The end-of-study survey included 9 questions assessing subjective burden (eg, feeling overwhelmed or annoyed; see Table 2).

### Analytic Plan

Compliance was evaluated using mixed effects regression models. A random intercept-only model was used to examine the proportion of variance in compliance rates attributable to individuals (ie, intraclass correlation coefficient [ICC]); a second model, including study week, was used to examine the impact of time in the study [55]. A similar approach was used to calculate the ICC in each of the latent variables and adaptive survey length.

### Adaptive Survey Length

**Figure 1.**
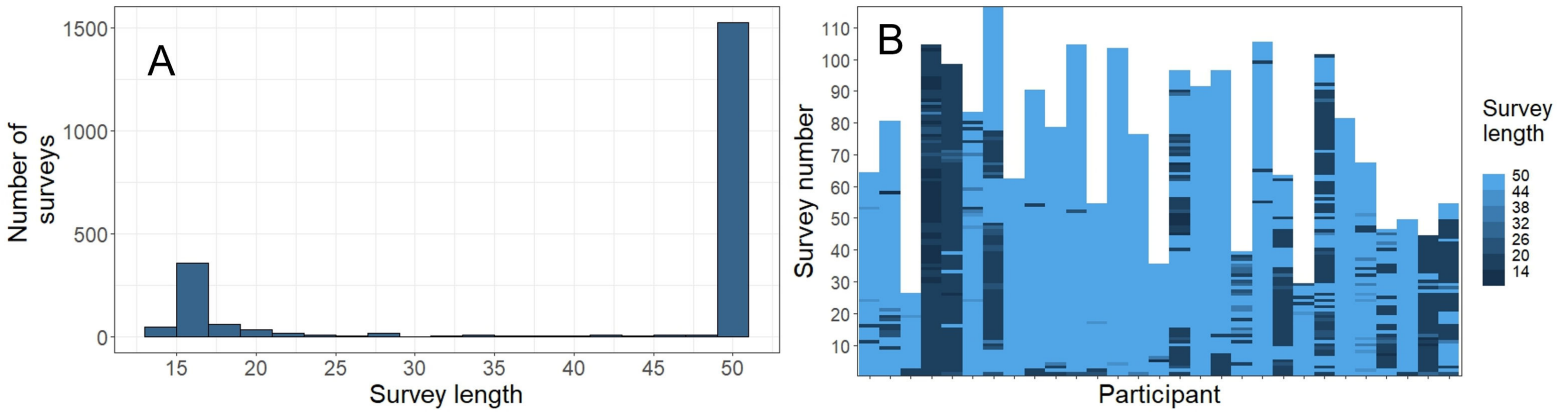
Adaptive survey length. (A) Histogram of survey lengths across the full study. (B) Survey length by participant over the study duration.

Survey length was examined further using a mixed effects model. Due to bimodality in survey lengths (see Figure 1), a binary variable where “1” denotes a survey of maximum length (ie, 50 items; n=1524) and “0” denotes a survey that ended before maximum length (n=610) was examined using mixed-effects logistic regression. CAT-SRP estimates and the number of surveys completed were included as fixed effects; a random slope for the number of surveys completed was also included to allow for potential within-person variability. Due to convergence issues upon inclusion of the random slope, Bayesian methods were adopted using the brms package [56] with default noninformative priors; odds ratios (OR) of fixed effects with 95% credible intervals excluding a null effect are reported.

### Adaptive Survey Content Overlap

To quantify item overlap, the Jaccard Index and overlap coefficient were calculated separately for each participant for all pairwise combinations of EMA surveys. For any 2 surveys, the Jaccard Index captures the ratio between the size of the intersection of items (ie, items in both surveys) and the union of items. It can be interpreted as the proportion of shared items (ie, between 2 surveys) to the total unique items across both surveys. The overlap coefficient is similar; however, the number of items for the shorter survey is used instead of the union. It can be interpreted as the proportion of shared items present in the shorter survey (ie, if survey lengths differ). Both metrics range from 0 to 1, with larger values suggesting more overlap in survey content. The average of these pairwise metrics was computed to provide a measure of survey similarity for each participant across their time in the study.

### CAT-SRP Factor Structure

The within- and between-person factor structure of CAT-SRP suicidal ideation items was assessed separately in study 2, using a 2-stage estimation process. First, the within- and between-person covariance matrices were estimated using pairwise complete observations with the weighted least squares estimator in Mplus (Version 8; Muthén & Muthén) [57]. Then parallel analysis was conducted on the resulting covariance matrices to explore dimensionality. Within- and between-person factor structures of CAT-SRP risk items were not explored due to small sample size, large proportion of missing item-level data, and limited variability in item responses (ie, within individuals). A multilevel EFA using Bayesian estimation in Mplus was attempted but never converged. The large degree of item-level missingness also prevented successful estimation with the 2-stage approach applied to STB items. The within- and between-person factor structures of the CAT-SRP domain scores are also explored.

### Concurrent and Predictive Validity

To assess the relationships between each of the assessed constructs and both passive and active suicidal ideation, multilevel models were fit to examine the concurrent and temporal relationships. Models included random intercepts to account for within-person clustering of observations. The concurrent model examined associations between suicidal ideation and psychological variables at the same time point. The lagged model investigated whether psychological variables predicted changes in suicidal ideation at the subsequent time point, controlling for current suicidal ideation. For prospective models, CAT-SRP scores from each completed survey were regressed onto scores from the previous completed survey using restricted maximum likelihood estimation. Fixed effects and marginal *R*^2^ statistics are reported.

### Subjective Burden

Descriptive statistics were calculated to evaluate subjective burden and acceptability of the study procedures (see Table 2 for subjective burden items).

### Ethical Considerations

All study procedures were reviewed and approved by the University of Notre Dame Institutional Review Board (protocol number 23-11-8206). Participants provided written and verbal informed consent prior to study procedures. Participants were informed that they were free to discontinue participation at any time by emailing study staff. All study data were deidentified and stored on secure servers to preserve confidentiality. Participants were compensated up to US $195 (see procedures for compensation details).

## Results

### Study 1

#### Study 1 Results Overview

**Figure 2.**
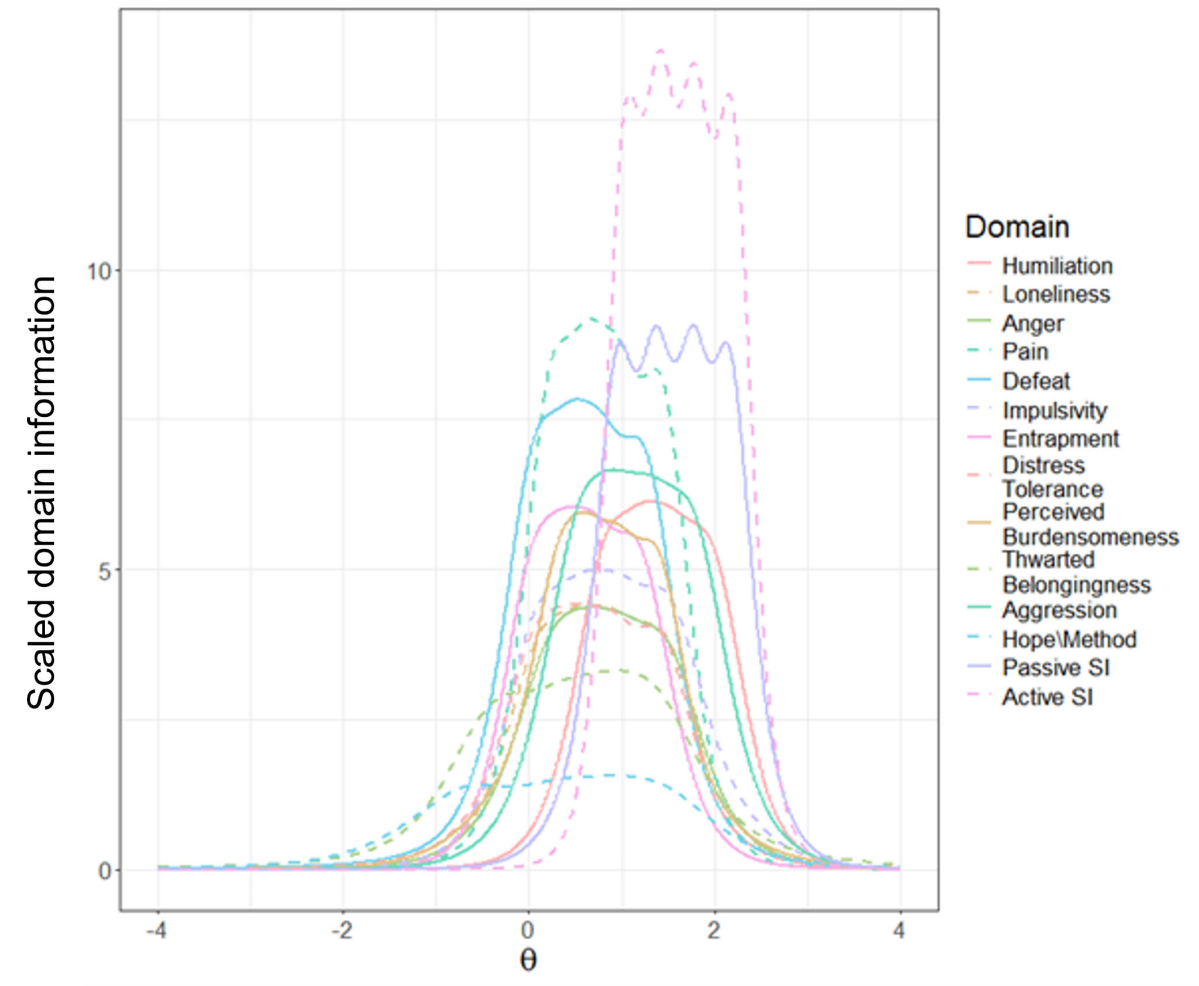
Computerized Adaptive Test of Suicide Risk Pathways domain information. Test information for each domain is divided by number of primary items in the item pool. SI: suicidal ideation.

To facilitate adaptive testing, a confirmatory MGRM was fit to the CAT-SRP risk items and J=18 CAT-SRP suicidal ideation items separately. Fit indices for the CAT-SRP risk model were unable to be computed due to model dimensionality. Interfactor correlations are provided in Table 3. Discrimination and difficulty parameters were extracted. Unidimensional domain information across levels of latent scores is presented in Figure 2A.

**Table 3.** CAT-SRP^a^ risk domain factor correlations.

Domain	Hum^b^	Lnly^c^	Ang^d^	Pain^e^	NU^f^	Dft^g^	Trap^h^	DT^i^	PB^j^	TB^k^	Hope	Agg^l^
Hum, *r*	1	0.56	0.561	0.441	0.478	0.487	0.54	0.58	0.541	0.16	0.051	0.383
Lnly, *r*	0.56	1	0.611	0.612	0.415	0.739	0.733	0.6	0.695	0.359	0.253	0.316
Ang, *r*	0.561	0.611	1	0.458	0.588	0.581	0.62	0.617	0.532	0.179	0.094	0.443
Pain, *r*	0.441	0.612	0.458	1	0	0.674	0.66	0.573	0.612	0.245	0.234	0.394
NU, *r*	0.478	0.415	0.588	0	1	0.336	0.373	0.457	0.407	0.072	0.023	0.252
Dft, *r*	0.487	0.739	0.581	0.674	0.336	1	0.78	0.62	0.683	0.334	0.355	0.299
Trap, *r*	0.54	0.733	0.62	0.66	0.373	0.78	1	0.649	0.713	0.335	0.288	0.335
DT, *r*	0.58	0.6	0.617	0.573	0.457	0.62	0.649	1	0.609	0.129	0.068	0.349
PB, *r*	0.541	0.695	0.532	0.612	0.407	0.683	0.713	0.609	1	0.342	0.291	0.439
TB, *r*	0.16	0.359	0.179	0.245	0.072	0.334	0.335	0.129	0.342	1	0.646	0.144
Hope, *r*	0.051	0.253	0.094	0.234	0.023	0.355	0.288	0.068	0.291	0.646	1	0.022
Agg, *r*	0.383	0.316	0.443	0.394	0.252	0.299	0.335	0.349	0.439	0.144	0.022	1

a CAT-SRP: Computerized Adaptive Test of Suicide Risk Pathways.

b Hum: humiliation.

c Lnly: loneliness.

d Ang: anger.

e Pain: psychological pain.

f NU: negative urgency impulsivity.

g Dft: defeat.

h Trap: entrapment.

i DT: distress tolerance.

j PB: perceived burdensomeness.

k TB: thwarted belongingness.

l Agg: aggression.

For the CAT-SRP suicidal ideation items, both the one-factor (root-mean-squared error of approximation [RMSEA]=0.048; Tucker Lewis Index [TLI]=0.991; Comparative Fit Index [CFI]=.0993) and 2-factor (RMSEA=0.053; TLI=0.989; CFI=0.991) graded response models exhibited good fit. As expected, passive and active suicidal ideation factors were highly correlated (*r*=0.92). Given the CAT-SRP’s overarching aim to study dynamics of suicidal thoughts and behaviors and prior work supporting differences between passive and active suicidal ideation [45,58], the 2-factor model was selected to calibrate the suicidal ideation items. Item discrimination and difficulty parameters were extracted for use in adaptive testing. Domain information is shown in Figure 1B. The final CAT-SRP item bank consists of J=189 risk items across 12 risk domains and J=18 suicidal ideation items split equally across passive and active ideation.

#### CAT-SRP Risk Factor Dimensionality

Parallel analysis suggested *M*=13 factors (see Figure S2 in Multimedia Appendix 1). Since 12 test domains were originally proposed for the CAT-SRP risk variables, EIFA was fit for both 12 and 13 factors to evaluate the interpretability of the resulting factor structures. Factor loading patterns are provided in Tables S1 and S2 in Multimedia Appendix 1. Results suggested that “perceived stress” items did not load onto a unique factor in either the 12- or 13-factor model. Thus, stress items were removed from the item pool, and the reduced pool was reanalyzed using EIFA. Items with positively valenced prompts, primarily from the hopelessness pool, all loaded onto a separate factor in both models (ie, after reverse coding), which is referred to as the Hope factor.

A series of EIFA models (M=10‐12) were fit to the reduced item pool; factor loading matrices are provided in Tables S3, S4, and S5 in Multimedia Appendix 1. The 12 model (Table S3 in Multimedia Appendix 1) was ultimately selected due to the interpretability of the factors, the lowest model BIC (BIC_M=12_*=*9384.88, BIC_M=11_*=*13967.56*,* BIC_M=10_=18452.14), and conceptual alignment with the hypothesized blueprint. This resulted in J=189 risk domain items. A summary of the final CAT-SRP risk domains and associated items is presented in Table 4.

**Table 4.** Computerized Adaptive Test of Suicide Risk Pathways risk item domain summary.

Domain	Total Items^a^ (Primary^b^), n (%)	Items
Humiliation	32 (32)	HUM^c^ 1‐32
Loneliness	30 (30)	INQ^d^ 11, 12, UCLA^e^ 1‐20, TBS^f^ 1‐8
Anger	24 (22)	Anger^g^ 1‐22, Impulse^h^ 11, Agg^i^ 1
Pain	16 (13)	Pain^j^ 1‐13, TRAP^k^ 3, 4, 7
Defeat	24 (14)	STHS^l^ 1, 2, 4, 5, 8, 10, DS^m^ 1‐8, DS 10‐16, BH.N^n^ 1‐3
Impulse (NU)	14 (11)	Impulse 1‐10, 12, Agg2 – 4
Entrapment	16 (16)	TRAP 1‐16
Distress tolerance	14 (14)	DT^o^ 1‐5, 7‐15
Perceived burdensomeness	17 (14)	INQ 1‐6, 11, 12, STHS 1, 2, 4, 5, 8, TBS 3, 7, Agg 2, 3
Thwarted belongingness	13 (7)	INQ 7‐10, 13‐15, TBS 3, 4, 8, DS 9, Impulse 11, DT 6
Aggression	6 (4)	Agg 1‐4, HUM 1, 2
Hope	12 (12)	STHS 3, 6, 7, 9, DS 2, 4, 9, Impulse 11, DT 6, BH.P^p^ 1‐3

a Some items load onto multiple factors.

b Primary items refer to how many items had their stronges factor loading on each domain.

c HUM: Humiliation Inventory.

d INQ: Interpersonal Needs Questionnaire.

e UCLA: UCLA (University of California, Los Angeles) Loneliness Scale.

f TBS: Thwarted Belongingness Scale.

g Anger: PROMIS (Patient-Reported Outcomes Measurement Information System) Anger.

h Impulse: UPPS-P (Urgency, (Lack of) Premeditation, (Lack of) Perseverance, Sensation Seeking, and Positive Urgency) Negative Urgency.

i Agg: PROMIS Aggression.

j Pain: psychological pain.

k TRAP: entrapment.

l STHS: State-Trait Hopelessness Scale.

m DS: Defeat Scale.

n BH.N: Brief Hopelessness – Negative.

o DT: Distress Tolerance Scale.

p BH.P: Brief Hopelessness – Positive.

To provide descriptive statistics for raw domain scores, sum scores were computed for each risk domain; items that loaded onto multiple factors were only included in sum scores for the domain with their strongest loading. Descriptive statistics for these domain-level sum scores are provided in Table 5. Internal consistency ranged from *α*=.90 to *α*=.99.

**Table 5.** Computerized Adaptive Test of Suicide Risk Pathways domain sum score descriptive statistics.

Domain	Mean (SD)		Min^a^-Max^b^	α	ω_h_	ω_total_	ICC^c^^,^^d^
Humiliation	61.07 (35.81)		32‐160	.99	0.90	0.99	0.49
Loneliness	67.48 (35.52)		30‐150	.99	0.91	0.99	0.57
Anger	47.55 (24.21)		22‐110	.97	0.89	0.98	0.39
Pain^e^	27.50 (16.13)		13‐65	.98	0.93	0.98	0.58
Defeat	31.46 (17.20)		14‐70	.98	0.94	0.98	0.55
Impulsivity	23.24 (12.17)		11‐55	.96	0.90	0.96	0.49
Entrapment	35.76 (19.51)		16‐80	.98	0.90	0.98	0.54
Distress Tolerance	32.02 (15.07)		14‐70	.96	0.75	0.96	0.53
PB^f^	30.69 (15.65)		14‐70	.96	0.85	0.96	0.63
TB^g^	16.58 (7.64)		7‐35	.91	0.86	0.92	0.54
Aggression	7.06 (4.22)		4‐20	.90	0.87	0.92	0.61
Hope	31.50 (11.04)		12‐60	.90	0.77	0.91	0.56
Passive SI^h^	21.88 (12.32)		9‐45	.98	0.93	0.98	0.55
Active SI	18.64 (11.612)		9‐45	.98	0.94	0.98	0.47

a Min: minimum observed score.

b Max: maximum observed score.

c computed in study 2.

d ICC: intraclass correlation.

e Pain: psychological pain.

f PB: perceived burdensomeness.

g TB: thwarted belongingness.

h SI: suicidal ideation.

### Study 2

#### Technical Issues

**Figure 3.**
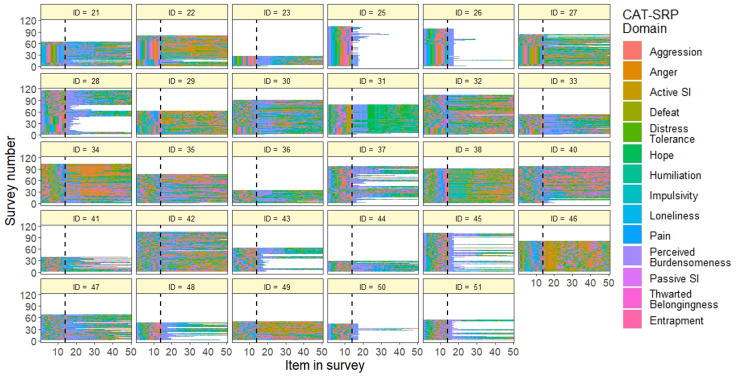
Computerized Adaptive Test of Suicide Risk Pathways survey content by participant over study duration. Dashed line=end of “warm-up” period. CAT-SRP: Computerized Adaptive Test of Suicide Risk Pathways; SI: suicidal ideation.

There were some technical difficulties with the web-based CAT-SRP system early in the study that led to some issues with data collection. At the beginning of data collection, there was an issue with the “warm-up” period, which led to respondents receiving the same first 14 items at the beginning of each survey (see also Figure 3B). Even though subsequent items were administered adaptively, this may have increased survey lengths early on. There was also an issue with the EMA survey alert system sent by the CAT-SRP, which failed to send additional future survey notifications if a participant did not respond to 2 consecutive surveys. This likely led to lower compliance rates for participants at the beginning of the study. Finally, the CAT-SRP did not explicitly record the number of survey nonresponses in the system. Instead, compliance was calculated as the ratio of surveys completed to the expected number of total surveys over the study period (n=126). This likely contributed to underestimation of compliance rates because of technical difficulties noted in the survey alert system.

#### Compliance

Compliance was calculated as the proportion of surveys completed over the expected number of total surveys over the study period (n=126). Overall compliance rates ranged from 20.63% to 83.33% (mean 55.88%, SD 19.38%), resulting in 2134 unique observations across the 29 participants. Compliance rates across study weeks are shown in Table S6 in Multimedia Appendix 1. The ICC for the random-intercept–only model suggested notable within-person variability in compliance rates (ICC=0.53). Consistent with prior work [55], compliance decreased with weeks in the study (b=−0.10; *P*<.001).

#### Adaptive Survey Length

Survey length showed a clear bimodal distribution (Figure 1A) with a large peak at 50 items and a smaller peak at 17 items. The random-intercept–only model suggested roughly half of

survey length variability was attributable to within-person factors (ICC=0.569). Figure 1B depicts person-level survey length over the duration of the study. Mixed-effect logistic regression analyses found that higher levels of humiliation (OR 0.72, 95% CI 0.59-0.87), impulsivity (ie, negative urgency; OR 0.81, 95% CI 0.69-0.96), aggression (OR 0.79, 95% CI 0.69-0.90), and active suicidal ideation (OR 0.61, 95% CI 0.46-0.80) were associated with reduced odds of surveys with maximum length; perceived burdensomeness levels (OR 1.12, 95% CI 1.01-1.25) increased the odds of maximum-length surveys (see Table 6).

**Table 6.** Bayesian logistic mixed-effects model of survey length.

	Estimate	SE	OR^a^ (95% CI)^b^	
Fixed effects				
Intercept	1.13	0.49	3.08 (1.16 to 7.95)	
Humiliation	*–0.33* ^c^	*0.09* ^c^	*–0.72*^c^ *(0.59 to 0.87*^c^*)*	
Loneliness	0.10	0.08	–1.10 (0.95 to 1.28)	
Anger	–0.12	0.09	–0.89 (0.75 to 1.06)	
Pain^d^	0.08	0.12	1.08 (0.86 to 1.36)	
Impulsivity	*–0.21* ^c^	*0.08* ^c^	*0.81*^c^ *(0.69 to 0.96*^c^*)*	
Defeat	0.07	0.10	1.07 (0.88 to 1.30)	
Entrapment	–0.06	0.08	0.94 (0.81 to 1.10)	
DT^e^	–0.16	0.10	0.85 (–0.36 to 0.03)	
PB^f^	*0.12* ^c^	*0.05* ^c^	*1.12*^c^ *(1.01 to 1.25*^c^*)*	
TB^g^	0.12	0.10	1.13 (0.93 to 1.35)	
Hope	–0.04	0.07	0.96 (0.84 to 1.11)	
Aggression	*–0.23* ^c^	*0.07* ^c^	*0.79*^c^ *(0.69 to 0.90*^c^*)*	
Passive SI^h^	–0.21	0.14	0.81 (0.62 to 1.07)	
Active SI	*–0.50* ^c^	*0.14* ^c^	*0.61*^c^ *(0.46 to 0.*^c^*80)*	
Surveys completed	0.03	0.02	1.03 (1.01 to 1.07)	
Random effects			95% CI	
SD (Intercept)	2.20	0.43	1.50 to 3.20	
SD (SC)^i^	0.06	0.02	0.04 to 0.11	
Cor^j^ (Intercept, SC)	**–**0.09	0.24	-0.54 to 0.38	

a OR: odds ratio.

b Bulk and tail effective sample sizes>1155.

c Credible interval for OR excludes 1.

d Pain: psychological pain.

e DT: distress tolerance.

f PB: perceived burdensomeness and hopelessness.

g TB: thwarted belongingness.

h SI: suicidal ideation.

i SC: surveys completed.

j Cor: correlation.

#### Adaptive Survey Content

After accounting for imbalanced item pool sizes, aggression, psychological pain, thwarted belongingness, passive suicidal ideation, and active suicidal ideation domains were administered more frequently (ie, compared to random administration); all other domains were administered less frequently. Figure 3 also suggests that the domain content of adaptive EMA surveys varied across participants (see also Figure S3 in Multimedia Appendix 1). The most and least frequently administered items from each domain are reported in Table 7. The most administered item (67.0% of surveys) was “Since the last prompt, my feelings of distress were so intense that they completely took over,” and the least administered item (2.8% of surveys) was “Since the last prompt, I felt isolated.” At the individual level, the most times an item was administered was 114 times, which corresponded to the item being present in 98.3% of that participant’s EMA surveys. Nine respondents had items that appeared in every survey (median 1, IQR 1-2). Conversely, the minimum number of times that an item was administered to an individual was one (median 20, IQR 7-24).

**Table 7.** Most and least common items by Computerized Adaptive Test of Suicide Risk Pathways domain.

Domain	Item prompts^a^	Values, n (%)^b^
Most common		
Active SI^c^	I wanted to die	1343 (62.9)
Aggression	I thought I may hit another person if I was provoked enough	1252 (58.7)
Anger	I had trouble controlling my anger	1251 (58.6)
Defeat	I felt that I have given up	960 (45)
DT^d^	My feelings of distress were so intense that they completely took over^h^	1429 (67)
Hope	I believe that I can help improve things	1158 (54.3)
Humiliation	I feared being ridiculed	997 (46.7)
Impulsivity	I often made worse because I acted without thinking when I was upset	1262 (59.1)
Loneliness	I felt completely alone	891 (41.8)
Pain^e^	I felt that my psychological pain was making me fall apart	989 (46.3)
Passive SI^c^	I thought that life was not worth living	1237 (58)
PB^f^	I felt that it was impossible to make progress on the goals I would like to strive for	1048 (49.1)
TB^g^	I interacted with people who care about me	1210 (56.7)
Entrapment	I felt that I was in a deep hole that I could not get out of	1012 (47.4)
Least common		
Active SI	I wanted to kill myself	617 (28.9)
Aggression	I have threatened people that I know	184 (8.62)
Anger	I held grudges toward others	278 (13)
Defeat	I felt that I sunk to the bottom of the ladder	323 (15.1)
DT	When I felt distressed or upset, I could not help but concentrate on how bad the distress actually feels	178 (8.34)
Hope	The future seems to be hopeful for me	216 (10.1)
Humiliation	I was concerned about being treated as invisible	141 (6.61)
Impulsivity	I got involved with things that I later wished I could get out of	187 (8.76)
Loneliness	I felt isolated^i^	59 (2.76)
Pain	I felt psychological pain	268 (12.6)
Passive SI	I wished I could go to sleep and never wake up	502 (23.5)
PB	I thought I make things worse for the people in my life	165 (7.73)
TB	I felt like I belong	363 (17)
Entrapment	I wanted to escape from my thoughts and feelings	105 (4.92)

a All items prompts preceded by “Since the last survey, …”.

b percentage refers to the percentage of EMA surveys which contained that item.

c SI: suicidal ideation.

d DT: distress tolerance.

e Pain: psychological pain.

f PB: perceived burdensomeness.

g TB: thwarted belongingness.

h Most frequently administered item.

i Least frequently administered item.

Average pairwise Jaccard Indices and overlap coefficients showed low to moderate overlap (median 0.35, IQR 0.27-0.45; median 0.37, IQR 0.31-0.49 respectively). Within-person average pairwise Jaccard Indices and overlap coefficients for each participant (ie, averaged over EMA surveys) ranged across participants (Jaccard Index=0.20‐0.77; overlap coefficient=0.25‐0.86); full summary data are provided in Table S7 in Multimedia Appendix 1. Thus, the adaptive nature of the CAT-SRP reduces the repetitiveness of EMA survey content relative to traditional EMA.

#### CAT-SRP Within- and Between-Person Structures

**Figure 4.**
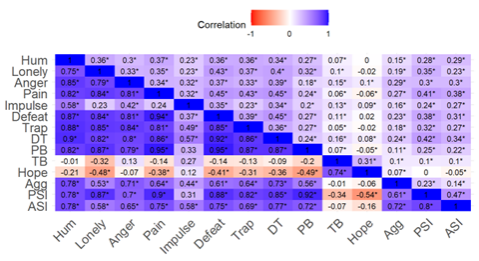
Computerized Adaptive Test of Suicide Risk Pathways domain within- and between-person correlation matrix. The upper triangle denotes within-person correlation, and the lower triangle denotes between-person correlation. Agg: aggression; ASI: active suicidal ideation; CAT-SRP: Computerized Adaptive Test of Suicide Risk Pathways; DT: distress tolerance; Hum: humiliation; Impulse: impulsivity; Pain: psychological pain; PB: perceived burdensomeness; PSI: passive suicidal ideation; TB: thwarted belongingness; Trap: eEntrapment.

Within- and between-person correlation matrices for the CAT-SRP domain scores over the EMA period are shown in Figure 4. In general, CAT-SRP domains were highly positively correlated between persons, except for the hope domain, which was negatively correlated, and the thwarted belongingness domain, which was uncorrelated with other domains. Within-person domains were moderately or weakly positively correlated with hope and thwarted belongingness, demonstrating the weakest correlations.

**Figure 5.**
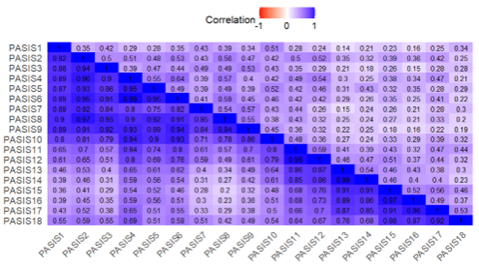
Computerized Adaptive Test of Suicide Risk Pathways item within- and between-person correlation matrix. The upper triangle denotes within-person correlation, and the lower triangle denotes between-person correlation.

Visual inspection of the CAT-SRP item response polychoric correlation matrices shows high positive between-person interitem correlations with items correlating higher within passive or active ideation domains and moderately between domains (Figure 5). Within-person polychoric correlations are lower in magnitude but appear to show a similar structure. To further explore these structures, we conducted parallel analysis, which suggested a 2-factor between-person and 1-factor within-person structure. Overall, however, between-person and within-person structures should be interpreted with caution given the small sample size.

#### Risk Factor Domain Associations

ICCs for risk factors ranged from 0.39 for anger to 0.63 for perceived burdensomeness, meaning that the between- versus within-person variability was nontrivial and roughly similar across each of the latent variables (Table 5). Relationships between risk factors and suicidal ideation were assessed concurrently (ie, same survey) and prospectively (ie, next survey; see Table 8). CAT-SRP risk domains explained 52.01% of concurrent passive suicidal ideation variance (*R*^2^=.521) with fixed effects of anger, psychological pain, defeat, entrapment, and perceived burdensomeness. Additionally, psychological pain, distress tolerance, and passive suicidal ideation demonstrated fixed effects on prospective passive suicidal ideation (*R*^2^=.301; *R*^2^=.184 when autoregressive effect is excluded). CAT-SRP risk domains also explained concurrent active suicidal ideation (*R*^2^=.392) with humiliation, anger, psychological pain, negative urgency impulsivity, defeat, distress tolerance, perceived burdensomeness, thwarted belongingness, and the method factor (ie, hope) demonstrating fixed effects. Prospective active suicidal ideation (*R*^2^=0.16; *R*^2^=0.093 when autoregressive effect is excluded) was associated with the method factor, distress tolerance, loneliness, and active suicidal ideation.

**Table 8.** Concurrent and prospective SI^a^ model results.

Concurrent models	Passive SI				Active SI			
Predictor	B*^b^*	SE	*t* test (*df*)	*P* value	B^b^	SE	*t* test (*df*)	*P* value
Intercept	0.34	0.11	2.96 (29.05)	.006	0.18	0.13	1.41 (30.57)	.17
Humiliation	0.02	0.02	0.95 (2130.69)	.34	*0.07*	*0.02*	*3.95 (2127.52)*	*<.001*
Loneliness	*0.07* ^*c*^	*0.02*	*4.36 (2133.67)*	*<.001*	–0.02	0.02	–1.52 (2133.45))	.12
Anger	*0.03*	*0.02*	*1.97 (2124.38)*	*.05*	*0.08*	*0.02*	*4.81 (2121.49)*	*<.001*
Pain	*0.15*	*0.02*	*7.50 (2131.28)*	*<.001*	*0.15*	*0.02*	*7.48 (2133.99)*	*<.001*
Impulsivity	0.01	0.02	0.63 (2031.07)	.53	*0.06*	*0.02*	*3.95 (2090.20)*	*<.001*
Defeat	*0.08*	*0.02*	*4.19 (2122.25)*	*<.001*	*0.05*	*0.02*	*2.45 (2119.73)*	*.02*
Entrapment	*0.04*	*0.02*	*2.44 (2131.51)*	*.01*	0.02	0.02	1.36 (2128.32)	.18
DT^d^	*0.14*	*0.02*	*7.97 (2128.69)*	*<.001*	*0.09*	*0.02*	*5.08 (2125.53)*	*<.001*
PB^e^	*0.05*	*0.01*	*3.76 (2119.83)*	*<.001*	*0.04*	*0.01*	*3.22 (2130.67)*	*0.001*
TB^f^	0.01	0.02	0.65 (2082.87)	.51	*0.05*	*0.02*	*2.72 (2115.04)*	*0.007*
Hope	–0.02	0.01	–1.33 (2109.46)	.18	*–0.06*	*0.01*	*–3.88 (2127.05)*	*<.001*
Aggression	*0.05*	*0.02*	*3.27 (2029.59)*	*.001*	–0.01	0.02	–0.42 (2088.77)	.68
**Prospective Models**								
Intercept	0.25	0.12	2.02 (25.86)	.05	0.18	0.13	1.34 (28.04)	.19
Lagged SI^g^	*0.32*	*0.02*	*13.36 (2104.97)*	*<.001*	*0.25*	*0.02*	*10.60 (2104.58)*	*<.001*
Humiliation	–0.01	0.02	–0.78 (2101.47)	.44	0.02	0.02	0.83 (2098.99)	.41
Loneliness	0.01	0.02	0.34 (2104.77)	.74	*–0.05*	*0.02*	*–2.66 (2104.98)*	*.008*
Anger	0.03	0.02	1.78 (2094.79)	.07	0.02	0.02	1.08 (2094.71)	.28
Pain	*0.05*	*0.02*	*2.14 (2103.81)*	*.03*	0.04	0.02	1.79 (2104.94)	.07
Impulsivity	–0.03	0.02	–1.62 (1981.51)	.10	–0.01	0.02	–0.30 (2040.26)	.76
Defeat	–0.02	0.02	–0.92 (2091.33)	.35	–0.00	0.02	–0.00 (2090.73)	>.99
Entrapment	0.02	0.02	1.18 (2101.63)	.24	–0.03	0.02	–1.40 (2100.56)	.16
DT^d^	*0.04*	*0.02*	*2.06 (2098.65)*	*.04*	*0.08*	*0.02*	*3.95 (2097.25)*	*<.001*
PB^e^	0.02	0.02	1.16 (2089.18)	.25	–0.00	0.02	–0.11 (2096.92)	.91
TB^f^	–0.03	0.02	–1.67 (2039.45)	.09	–0.00	0.02	–0.15 (2071.71)	.88
Hope	–0.02	0.02	–1.09 (2076.12)	.27	*–0.05*	*0.02*	*–3.19 (2089.90)*	*.001*
Aggression	0.03	0.02	1.72 (1983.10)	.09	0.01	0.02	0.58 (2035.33)	.56

a SI: suicidal ideation.

b Fixed effect regression coefficients.

c Italicized values indicate *P*<.05.

d DT: distress tolerance.

e PB: perceived burdensomeness.

f TB: thwarted belongingness.

g Lagged SI: auto-regressive effect of passive or active suicidal ideation.

#### Subjective Burden

Complete descriptive statistics for subjective burden items are provided in Table 2. Over 65% of participants reported little to no feeling of being overwhelmed with the number of survey questions. Over 75% of participants also reported little to no frustration or annoyance due to the number of survey questions. Additionally, 67.9% of participants indicated that they never skipped surveys because of the survey length, with 25% indicating that survey length contributed to them skipping occasional surveys (ie, less than one per week).

## Discussion

### Study 1

#### Study 1 Background

The present study calibrated the CAT-SRP, a multidimensional CAT for measuring suicide risk domains and suicidal ideation in intensive longitudinal designs. Initial risk domains were identified by literature review and consultation with experts. Exploratory factor analyses were conducted to empirically evaluate the dimensionality of the CAT-SRP risk item bank. Results suggested 12 risk domains, which, while largely consistent with those identified in theoretical models [2-4], somewhat deviated from the initial measure blueprint. Notable deviations from the anticipated domain structure included (1) aggression items split from anger items into a separate factor; (2) hopelessness items loaded onto defeat and PB factors rather than as a separate factor; and (3) a method factor for positively valenced item prompts emerged. This method factor was present in all models examined and was primarily indexed by items reflecting hope (ie, rather than hopelessness). Research suggests that hope and hopelessness are different constructs [59,60] and which have demonstrated different relationships regarding suicide risk [61] potentially explaining some of the above deviations. Additionally, after inspection of the item prompts, the TB domain captured in the CAT-SRP appears to reflect the absence of reciprocal care, while the loneliness domain captures social isolation. As anticipated, the CAT-SRP suicidal ideation domains include passive and active suicidal ideation.

Information curves suggest that most CAT-SRP items were best at capturing average to above-average levels of the corresponding domain severity (Figure 1). This was also the case for suicidal ideation items. Thus, CAT-SRP scores for these domains can be expected to effectively differentiate between respondents with elevated risk severity but less effectively differentiate between respondents exhibiting lower levels of risk. Exceptions to this include the humiliation, anger, and aggression domains, which exhibit more reliable measurement at below-average levels. CAT-SRP domains also differed in the number of items present in the item bank. Although most domains contained between 10‐20 items, the aggression domain only consisted of 6 items (4 primary). Conversely, the humiliation and loneliness domains consisted of 32 and 30 items, respectively. Domains with deeper item pools are more likely to demonstrate increased measurement precision across a wider range of severity levels and may result in less repetitive item administration in EMA contexts. Future work may seek to add items that broaden the domain coverage of the CAT-SRP item bank. Importantly, however, these findings suggest that the performance of suicide risk measures in intensive longitudinal data differs depending on the underlying level of risk severity.

#### Study 1 Limitations

Findings should be interpreted in the context of study limitations. First, although the CAT-SRP is intended for use in intensive longitudinal designs, the present calibration sample was collected using a large cross-sectional sample with day-level temporal referents. This approach was selected due to the large sample size needed to obtain sufficiently accurate MGRM item parameter estimates [50,62]. This precluded the evaluation of longitudinal measurement invariance. Additionally, item parameters obtained in this calibration study are for items with a day-level temporal referent. Thus, they are likely directly applicable to daily diary research. However, additional work is needed to determine if more granular temporal referents (eg, “Since the last prompt”) substantially impact item parameters. Future work should also seek to validate the 12-dimensional CAT-SRP structure observed in the current study.

It is also important to note that the sample used in this calibration study was a general adult sample collected through Prime Panels. Although prior research has supported the use of Prime Panels [63] and multiple efforts were taken to safeguard data quality, other work has highlighted potential pitfalls of online crowdsourcing data collection platforms [64]. Additionally, although only 36.33% of the calibration sample reported a lifetime history of suicidal thoughts and behavior, risk domains assessed by the CAT-SRP are applicable to individuals with or without a history of suicide risk. Further, to study the development of these risk pathways, it is necessary to measure risk domains among those who have yet to exhibit suicidal thoughts or behaviors. Finally, it is important to note that risk domains and items were selected based on predominant ideation-to-action theoretical models (ie, ITS, 3ST, IMV) and expert consultation given the preliminary nature of this study and not in direct collaboration with individuals with lived experiences. Future work will aim to refine and expand the CAT-SRP item bank to include input from those with lived experiences.

#### Study 1 Conclusions

The present study developed and calibrated the CAT-SRP in a large community sample, establishing a comprehensive measurement system that captures 12 empirically derived domains of suicide risk, along with passive and active suicidal ideation. The domain structure largely aligns with theoretical models while revealing some notable refinements, such as the distinction between anger and aggression domains, the distribution of hopelessness across multiple factors, and the emergence of a potential protective factor. Information analyses suggest the CAT-SRP provides optimal measurement precision at different severity levels across domains, potentially allowing for a more nuanced assessment of risk pathways. While the cross-sectional calibration provides a foundation for adaptive measurement, Study 2 represents a critical next step in evaluating the CAT-SRP’s utility for intensive longitudinal assessment in clinical populations.

### Study 2

#### Study 2 Background

The present pilot study implemented and evaluated an adaptive ecological momentary assessment protocol using the CAT-SRP among individuals with past-month suicidal thoughts or behaviors. Although prior work has shown promise for CAT in patient-reported outcomes measurement [22], this represents the first application of CAT for intensive longitudinal assessment of suicide risk factors and suicidal ideation and the first application of MCAT in EMA. Results support the feasibility and acceptability of the CAT-SRP for real-time assessment while providing rich information about the dynamics of suicide risk factors and ideation in daily life. One key finding was that the CAT-SRP captured both between- and within-person variability in risk factors and suicidal ideation. Intraclass correlations indicated that roughly half of the variance in most constructs was attributable to within-person fluctuations, supporting the dynamic nature of these processes. The adaptive testing algorithm showed variability in survey length and content across participants and occasions, suggesting personalized assessment. Although the overall surveys only showed moderate overlap on average, nine participants did receive a small subset of items (eg, a single item) in every survey. This was likely due to a lack of variability in item responses for a given domain. Future implementations of CAT in EMA designs that are concerned with survey repetitiveness may wish to directly incorporate content balancing in their selection algorithm [65]. Additionally, future versions of the CAT-SRP would benefit from directly tracking participant-level compliance.

**Figure 6.**
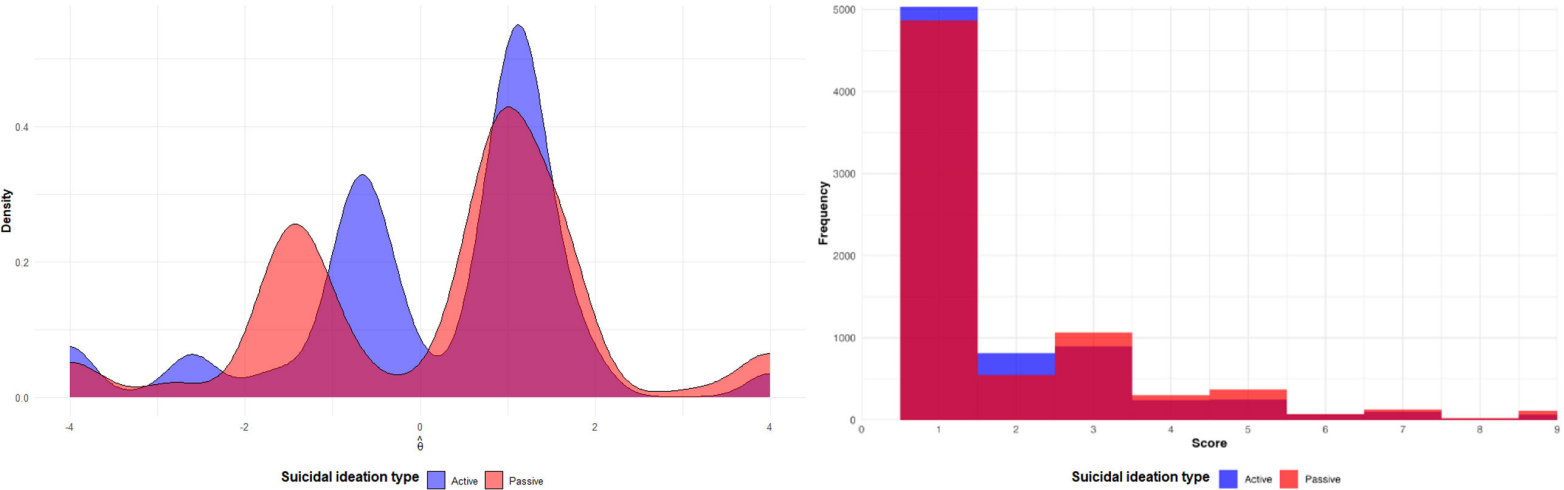
Distribution of active and passive suicidal ideation. The left plot is the density plot from the current study, while the right plot is a histogram from a similar study that used the sum of 2 items for both passive and active suicidal ideation [66].

Initial analysis of the relationships between CAT-SRP risk domain scores with passive and active suicidal ideation was also promising. Analyses of relationships between risk factors and passive ideation at the same measurement occasion (ie, concurrent effects) found effects for 8 of 12 risk factors; 9 of the 12 risk factors were found to relate to concurrent active ideation. Additionally, CAT-SRP risk domains accounted for 52% and 39% of variability in concurrent passive and active suicidal ideation domain scores and 18% and 9% of variability in prospective ideation, suggesting promising concurrent and predictive validity of CAT-SRP risk factor domain scores. Although future research is needed, these preliminary findings suggest that adaptive assessment of suicidal ideation and associated risk factors may alleviate measurement challenges common in EMA suicide research (ie, zero-inflation; see Figure 6) and enhance signal detection [14].

The implementation of adaptive assessment in this context has several important implications for advancing the study of suicide risk. First, the ability to efficiently measure multiple risk domains while maintaining measurement precision addresses a critical limitation of traditional EMA approaches that often rely on abbreviated measures. Second, assessing this breadth of risk factors has important implications for advancing suicide theory. Importantly, future research should consider pairing CAT-SRP with methods that also vary the timing of assessments, such as including more intensive windows of assessments [6]. When combined with recent developments in continuous time models, this approach can enhance our understanding of both the key risk factors and the timescales at which they exert the greatest influence [67,68].

Advancing theoretical understanding of clinical phenomena requires sophisticated, dimensional assessment approaches. While the current study examined 12 risk factors, this represents only a subset of potentially relevant constructs, particularly when considering the extant body of suicide research and when compared to comprehensive frameworks like the Hierarchical Taxonomy of Psychopathology [69]. Additionally, to fully capture the complexity of suicide risk, it is essential to incorporate protective factors [70], which the CAT-SRP currently lacks. This underscores a fundamental tension in EMA research: balancing comprehensive assessment with practical constraints and response burden. While the CAT-SRP aims to mitigate this issue, future research could address this challenge through innovative design approaches, such as planned missingness at the construct level or dynamic selection of relevant constructs based on individual risk profiles and temporal patterns. These methods could expand the breadth of assessment while maintaining feasibility for intensive longitudinal designs.

#### Study 2 Limitations

Although the current study highlights the potential benefits of the CAT-SRP for assessing suicidal ideation and associated risk factors in EMA, these findings should be interpreted in the context of the study’s limitations. First, it is important to reiterate that study 2 was a pilot study consisting of only 29 respondents, thus requiring replication in a larger sample. Additionally, the CAT-SRP used “since the last prompt” as the temporal referent for all items, which differed from the calibration referent (“Today”). This referent was chosen because of its suitability for EMA designs while remaining more consistent with the calibration referent than other referents common to EMA designs (eg, “right now”). Given heterogeneity in temporal dynamics in suicidal thinking and related processes [6], future work is likely needed to evaluate the potential impact of this choice on the risk factors assessed by the CAT-SRP.

Technical difficulties with the CAT-SRP web-based survey system study also introduced challenges in assessing study compliance. Compliance rates were calculated as the number of complete surveys over the number of expected surveys throughout the study period. However, technical difficulties early in the study led to (1) failure to send additional survey notifications if a participant did not respond to 2 consecutive surveys and (2) failure to directly record nonresponses in the CAT-SRP platform. Thus, compliance rates reported in the current study are likely underestimated. This initial version of the CAT-SRP was also unable to tailor the daily EMA window to the participant (ie, all respondents received surveys between 9 AM EST and 9 PM EST). Future versions of the CAT-SRP will address these limitations, which will likely bolster compliance rates.

Finally, while the CAT-SRP appeared to successfully tailor survey content, most surveys reached the maximum allowed length (ie, 50 items). Although these surveys were longer than some previous studies [58], the compliance rates were similar [55], and participants reported low levels of subjective burden. Thus, even for surveys that reach maximum length, there may be benefits to reducing the repetitiveness of EMA survey content. Since CAT tailors survey items to respondents based on estimated underlying levels of risk factors, the content validity of CAT-SRP surveys is hypothesized to be higher than static measures [71]; however, an interesting area for future work would be to examine the content validity of CAT-SRP item content for participants with different longitudinal risk profiles. Further methodological research is also needed to determine optimal stopping rules and item selection procedures that balance measurement precision with participant burden for intensive longitudinal designs. Some simulation work has suggested that sequential application of unidimensional stopping rules may increase efficiency in intensive longitudinal designs [53]. Additionally, alternative stopping rules, which use predicted SE reduction [72], may also increase precision with minimal impact on measurement precision [73]. Finally, it is important to note that since the CAT-SRP relies on a precalibrated item bank, measurement invariance is assumed for the duration of the EMA period. Further methodological research is needed to relax this assumption and account for the possibility that item parameters change over repeated exposure to the item (ie, item parameter drift) in adaptive EMA. This is particularly important for domains where repeatedly responding to certain items may influence the underlying relationship between items and domain (eg, reactivity [74]).

### General Conclusions

This study calibrated and piloted the CAT-SRP, the first multidimensional adaptive assessment of measuring suicidal thoughts and risk factors in EMA designs. While motivated by ideation-to-action theoretical models, the structure of risk factors was empirically derived in a large cross-sectional sample. Findings suggested that hopelessness items split across defeat and perceived burdensomeness domains rather than emerging as a distinct factor, potentially indicating that hopelessness may be better understood as an element of broader negative self-evaluative processes. Additionally, a method factor composed primarily of positively valenced items also raised questions about potential mixing of risk and protective constructs in existing assessment. In total, 12 risk factors were included in the CAT-SRP alongside passive and active suicidal ideation domains.

A pilot EMA study provided preliminary validation of the CAT-SRP in intensive longitudinal data. Results, although preliminary, suggest that the CAT-SRP produces dynamic survey content (ie, different items) over the course of an EMA study while maintaining measurement precision. This marks the first application of CAT in suicide EMA research and the first application of MCAT to intensive longitudinal designs and opens new possibilities for research design. The different patterns of measurement precision across domains—with some providing better discrimination at low levels and others at high levels—suggest the potential for developing targeted assessment strategies based on individual risk profiles. Future research could explore dynamically adapting assessment frequency and domain coverage, integrating passive sensing data, or linking adaptive measurement methodologies with just-in-time adaptive interventions.

## Supplementary material

10.2196/76544 Multimedia Appendix 1 Repetition to relevance.

## Abbreviations

3ST: Three-Step Theory of Suicide
CAT: Computerized Adaptive Testing
CAT-SRP: Computerized Adaptive Test of Suicide Risk Pathways
CAT-SS: Computerized Adaptive Test-Suicide Scale
CFI: Comparative Fit Index
EIFA: exploratory item factor analysis
EMA: ecological momentary assessment
ICC: intraclass correlation coefficient
IMV: Integrated Motivational-Volitional
INQ: Interpersonal Needs Questionnaire
ITS: Interpersonal Theory of Suicide
MGRM: multidimensional graded response model
OR: odds ratios
PASIS: passive and active suicidal ideation scale
PB: perceived burdensomeness
PROMIS: Patient-Reported Outcomes Measurement Information System
RMSEA: root-mean-square error of approximation
STB: suicidal thoughts and behavior
TB: thwarted belongingness
TLI: Tucker Lewis Index
UCLA: University of California, Los Angeles
UPPS-P: Urgency, (Lack of) Premeditation, (Lack of) Perseverance, Sensation Seeking, and Positive Urgency
