# Supplementary material for: Initial Development of a Multidimensional Computerized Adaptive Test for Intensive Longitudinal Assessment of Suicide Risk: Development and Usability Study
Source: JMIR Form Res. 2025 Nov 26;9:e76544. doi: 10.2196/76544 (PMC12661605; doi:10.2196/76544)
Supplement: Multimedia Appendix 1 [file formative-v9-e76544-s001.docx]

**Supplemental Materials: Repetition to Relevance**

**Appendix A: Supplemental Figures**

**Figure S1. Study 2 Flowchart**

**
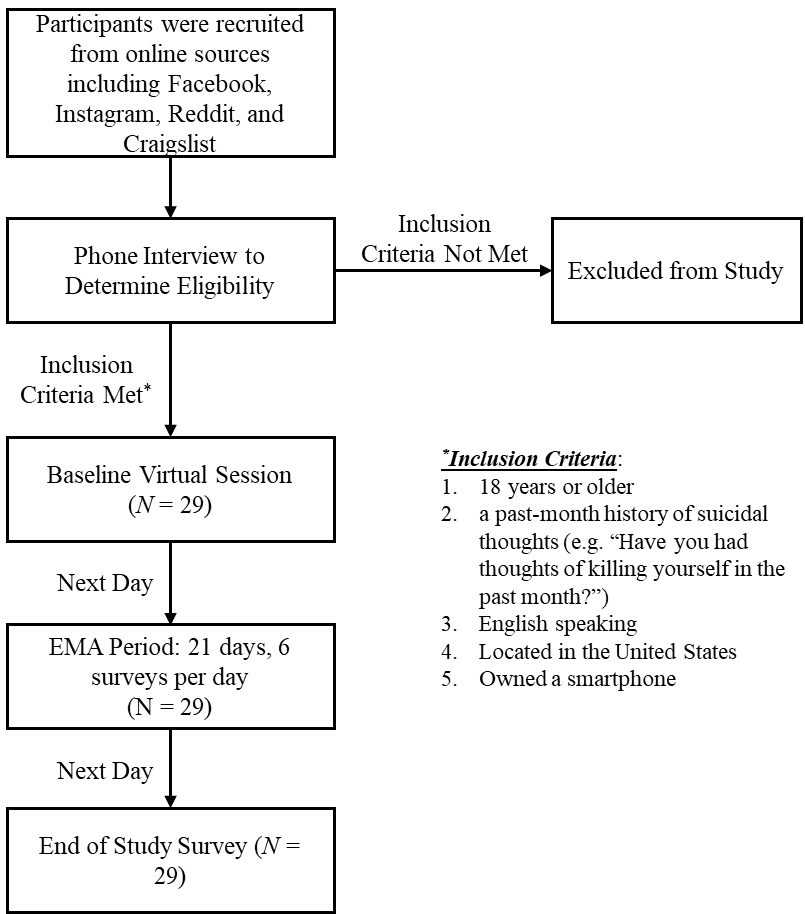
**

**Figure S2.** Parallel Analysis of CAT-SRP Polychoric Correlation Matrices


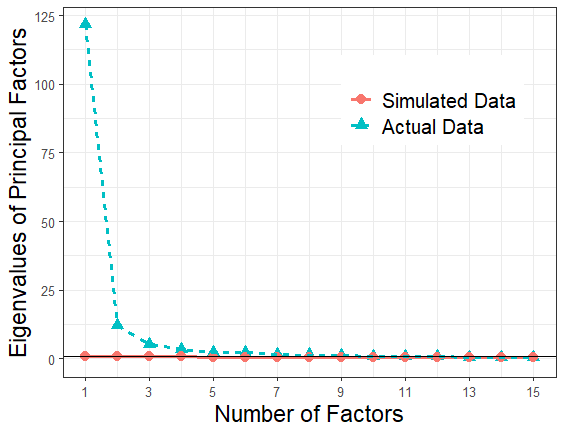


**Figure S3.** CAT-SRP Survey Domains by Participant

**
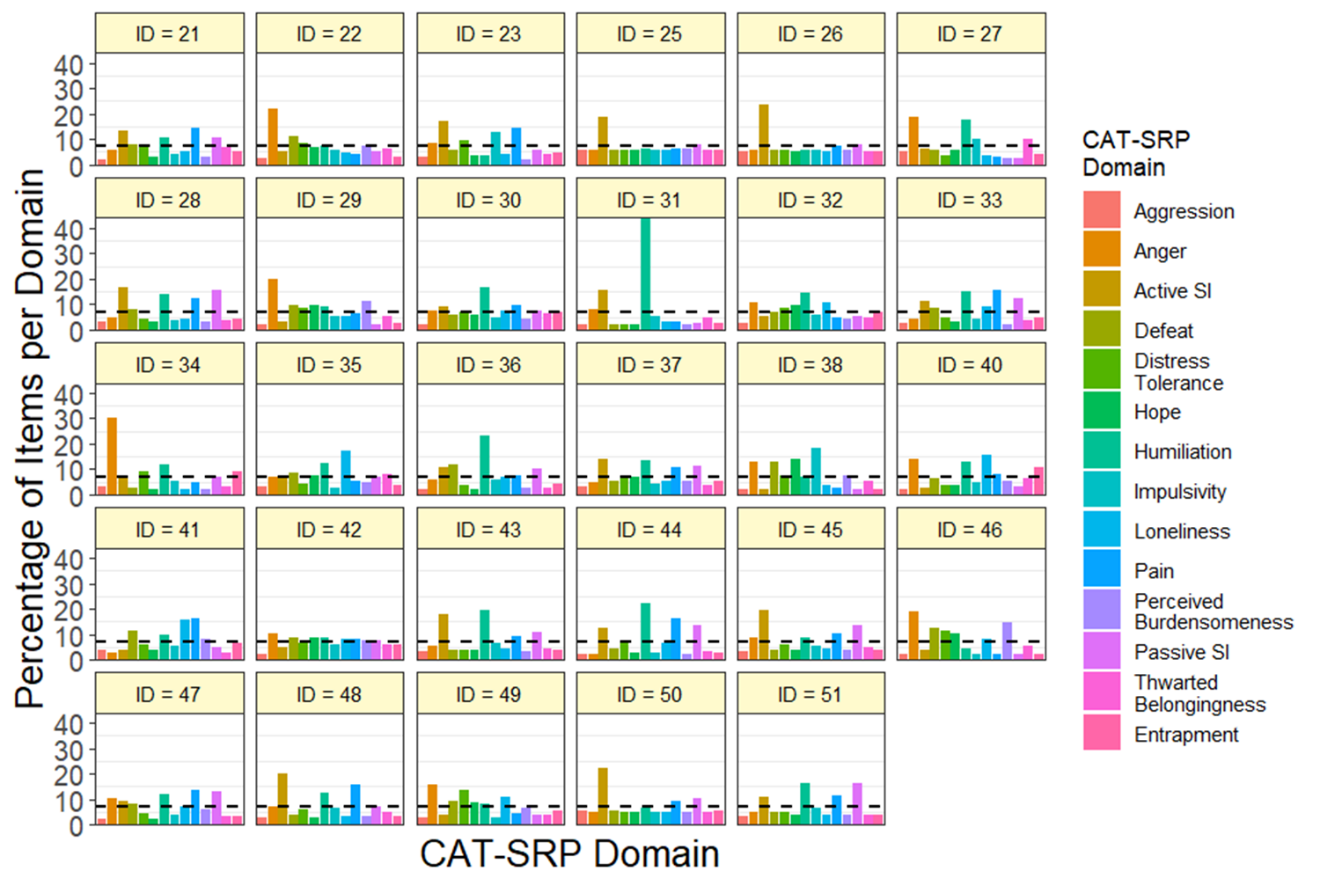
**

**Appendix B: Supplemental Tables**

**Table S1**. Factor Loading Patterns for 13-dimensional EIFA in Full Initial Item Pool.

|  | F1 | F2 | F3 | F4 | F5 | F6 | F7 | F8 | F9 | F10 | F11 | F12 | F13 |
| --- | --- | --- | --- | --- | --- | --- | --- | --- | --- | --- | --- | --- | --- |
| INQ1 | - | - | - | - | - | - | - | 0.64 | - | - | - | - | - |
| INQ2 | - | - | - | - | - | - | - | 0.6 | - | - | - | - | - |
| INQ3 | - | - | - | - | - | - | - | 0.59 | - | - | - | - | - |
| INQ4 | - | - | - | - | - | - | - | 0.68 | - | - | - | - | - |
| INQ5 | - | - | - | - | - | - | - | 0.58 | - | - | - | - | - |
| INQ6 | - | - | - | - | - | - | - | 0.54 | - | - | - | - | - |
| INQ7. | - | - | - | - | - | - | - | - | - | 0.73 | - | - | - |
| INQ8. | - | - | - | - | - | - | - | - | - | 0.68 | - | - | - |
| INQ9. | - | - | - | - | - | - | - | - | - | 0.82 | - | - | - |
| INQ10. | - | - | - | - | - | - | - | - | - | 0.79 | - | - | - |
| INQ11 | - | 0.31 | - | - | - | - | - | 0.26 | - | - | - | - | - |
| INQ12 | - | 0.27 | - | - | - | - | - | 0.28 | - | - | - | - | - |
| INQ13. | - | - | - | - | - | - | - | - | - | 0.82 | - | - | - |
| INQ14. | - | - | - | - | - | - | - | - | - | 0.79 | - | - | - |
| INQ15. | - | - | - | - | - | - | - | - | - | 0.75 | - | - | - |
| UCLA1 | - | 0.62 | - | - | - | - | - | - | - | - | - | - | - |
| UCLA2 | - | 0.7 | - | - | - | - | - | - | - | - | - | - | - |
| UCLA3 | - | 0.6 | - | - | - | - | - | - | - | - | - | - | - |
| UCLA4 | - | 0.81 | - | - | - | - | - | - | - | - | - | - | - |
| UCLA5 | - | 0.66 | - | - | - | - | - | - | - | - | - | - | - |
| UCLA6 | - | 0.63 | - | - | - | - | - | - | - | - | - | - | - |
| UCLA7 | - | 0.7 | - | - | - | - | - | - | - | - | - | - | - |
| UCLA8 | - | 0.69 | - | - | - | - | - | - | - | - | - | - | - |
| UCLA9 | - | 0.65 | - | - | - | - | - | - | - | - | - | - | - |
| UCLA10 | - | 0.68 | - | - | - | - | - | - | - | - | - | - | - |
| UCLA11 | - | 0.67 | - | - | - | - | - | - | - | - | - | - | - |
| UCLA12 | - | 0.61 | - | - | - | - | - | - | - | - | - | - | - |
| UCLA13 | - | 0.59 | - | - | - | - | - | - | - | - | - | - | - |
| UCLA14 | - | 0.64 | - | - | - | - | - | - | - | - | - | - | - |
| UCLA15 | - | 0.69 | - | - | - | - | - | - | - | - | - | - | - |
| UCLA16 | - | 0.71 | - | - | - | - | - | - | - | - | - | - | - |
| UCLA17 | - | 0.55 | - | - | - | - | - | - | - | - | - | - | - |
| UCLA18 | - | 0.59 | - | - | - | - | - | - | - | - | - | - | - |
| UCLA19 | - | 0.61 | - | - | - | - | - | - | - | - | - | - | - |
| UCLA20 | - | 0.58 | - | - | - | - | - | - | - | - | - | - | - |
| STHS1 | - | - | - | - | - | - | 0.23 | 0.26 | - | - | - | - | - |
| STHS2 | - | - | - | - | - | - | 0.25 | 0.27 | - | - | - | - | - |
| STHS3. | - | - | - | - | - | - | - | - | - | - | 0.68 | - | - |
| STHS4 | - | - | - | - | - | - | 0.23 | 0.27 | - | - | 0.22 | - | - |
| STHS5 | - | - | - | - | - | - | 0.23 | 0.25 | - | - | - | - | - |
| STHS6. | - | - | - | - | - | - | - | - | - | - | 0.68 | - | - |
| STHS7. | - | - | - | - | - | - | - | - | - | - | 0.83 | - | - |
| STHS8 | - | - | - | - | - | - | - | 0.36 | - | - | - | - | - |
| STHS9. | - | - | - | - | - | - | - | - | - | - | 0.73 | - | - |
| STHS10 | - | - | - | - | - | - | 0.28 | - | - | - | - | - | - |
| TBS1 | - | 0.32 | - | - | - | - | - | - | - | - | - | - | - |
| TBS2 | - | 0.29 | - | - | - | - | - | - | - | - | - | - | - |
| TBS3 | - | 0.23 | - | - | - | - | - | - | - | 0.23 | - | - | - |
| TBS4 | - | 0.31 | - | - | - | - | - | - | - | 0.23 | - | - | - |
| TBS5 | - | 0.26 | - | - | - | - | - | - | - | - | - | - | - |
| TBS6 | - | 0.32 | - | - | - | - | - | - | - | - | - | - | - |
| TBS7 | - | 0.34 | - | - | - | - | - | 0.21 | - | 0.2 | - | - | - |
| TBS8 | - | 0.37 | - | - | - | - | - | - | - | 0.21 | - | - | - |
| PB.VAS | - | - | - | - | - | - | - | 0.22 | - | - | - | - | - |
| DS1 | - | - | - | - | - | - | 0.5 | - | - | - | - | - | - |
| DS2. | - | - | - | - | - | - | 0.21 | - | - | - | 0.34 | - | - |
| DS3 | - | - | - | - | - | - | 0.51 | - | - | - | - | - | - |
| DS4. | - | - | - | - | - | - | 0.26 | - | - | - | 0.36 | - | - |
| DS5 | - | - | - | - | - | - | 0.47 | - | - | - | - | - | - |
| DS6 | - | - | - | - | - | - | 0.47 | - | - | - | - | - | - |
| DS7 | - | - | - | - | - | - | 0.52 | - | - | - | - | - | - |
| DS8 | - | - | - | - | - | - | 0.47 | - | - | - | - | - | - |
| DS9. | - | - | - | - | - | - | - | - | - | - | 0.28 | 0.33 | - |
| DS10 | - | - | - | - | - | - | 0.49 | - | - | - | - | - | - |
| DS11 | - | - | - | - | - | - | 0.47 | - | - | - | - | - | - |
| DS12 | - | - | - | - | - | - | 0.5 | - | - | - | - | - | - |
| DS13 | - | - | - | - | - | - | 0.43 | - | - | - | - | - | - |
| DS14 | - | - | - | - | - | - | 0.46 | - | - | - | - | - | - |
| DS15 | - | - | - | - | - | - | 0.49 | - | - | - | - | - | - |
| DS16 | - | - | - | - | - | - | 0.42 | - | - | - | - | - | - |
| TRAP1 | - | - | - | - | - | 0.37 | - | - | - | - | - | - | - |
| TRAP2 | - | - | - | - | - | 0.39 | - | - | - | - | - | - | - |
| TRAP3 | - | - | - | 0.21 | - | 0.45 | - | - | - | - | - | - | - |
| TRAP4 | - | - | - | 0.24 | - | 0.42 | - | - | - | - | - | - | - |
| TRAP5 | - | - | - | - | - | 0.47 | - | - | - | - | - | - | - |
| TRAP6 | - | - | - | - | - | 0.42 | - | - | - | - | - | - | - |
| TRAP7 | - | - | - | 0.21 | - | 0.46 | - | - | - | - | - | - | - |
| TRAP8 | - | - | - | - | - | 0.5 | - | - | - | - | - | - | - |
| TRAP9 | - | - | - | - | - | 0.49 | - | - | - | - | - | - | - |
| TRAP10 | - | - | - | - | - | 0.5 | - | - | - | - | - | - | - |
| TRAP11 | - | - | - | - | - | 0.51 | - | - | - | - | - | - | - |
| TRAP12 | - | - | - | - | - | 0.53 | - | - | - | - | - | - | - |
| TRAP13 | - | - | - | - | - | 0.51 | - | - | - | - | - | - | - |
| TRAP14 | - | - | - | - | - | 0.41 | - | - | - | - | - | - | - |
| TRAP15 | - | - | - | - | - | 0.51 | - | - | - | - | - | - | - |
| TRAP16 | - | - | - | - | - | 0.48 | - | - | - | - | - | - | - |
| HUM1 | 0.61 | - | - | - | - | - | - | - | - | - | - | - | - |
| HUM2 | 0.69 | - | - | - | - | - | - | - | - | - | - | - | - |
| HUM3 | 0.72 | - | - | - | - | - | - | - | - | - | - | - | - |
| HUM4 | 0.68 | - | - | - | - | - | - | - | - | - | - | - | - |
| HUM5 | 0.75 | - | - | - | - | - | - | - | - | - | - | - | - |
| HUM6 | 0.78 | - | - | - | - | - | - | - | - | - | - | - | - |
| HUM7 | 0.79 | - | - | - | - | - | - | - | - | - | - | - | - |
| HUM8 | 0.78 | - | - | - | - | - | - | - | - | - | - | - | - |
| HUM9 | 0.79 | - | - | - | - | - | - | - | - | - | - | - | - |
| HUM10 | 0.8 | - | - | - | - | - | - | - | - | - | - | - | - |
| HUM11 | 0.84 | - | - | - | - | - | - | - | - | - | - | - | - |
| HUM12 | 0.81 | - | - | - | - | - | - | - | - | - | - | - | - |
| HUM13 | 0.8 | - | - | - | - | - | - | - | - | - | - | - | - |
| HUM14 | 0.86 | - | - | - | - | - | - | - | - | - | - | - | - |
| HUM15 | 0.87 | - | - | - | - | - | - | - | - | - | - | - | - |
| HUM16 | 0.77 | - | - | - | - | - | - | - | - | - | - | - | - |
| HUM17 | 0.89 | - | - | - | - | - | - | - | - | - | - | - | - |
| HUM18 | 0.82 | - | - | - | - | - | - | - | - | - | - | - | - |
| HUM19 | 0.79 | - | - | - | - | - | - | - | - | - | - | - | - |
| HUM20 | 0.84 | - | - | - | - | - | - | - | - | - | - | - | - |
| HUM21 | 0.86 | - | - | - | - | - | - | - | - | - | - | - | - |
| HUM22 | 0.82 | - | - | - | - | - | - | - | - | - | - | - | - |
| HUM23 | 0.77 | - | - | - | - | - | - | - | - | - | - | - | - |
| HUM24 | 0.83 | - | - | - | - | - | - | - | - | - | - | - | - |
| HUM25 | 0.81 | - | - | - | - | - | - | - | - | - | - | - | - |
| HUM26 | 0.75 | - | - | - | - | - | - | - | - | - | - | - | - |
| HUM27 | 0.74 | - | - | - | - | - | - | - | - | - | - | - | - |
| HUM28 | 0.73 | - | - | - | - | - | - | - | - | - | - | - | 0.2 |
| HUM29 | 0.79 | - | - | - | - | - | - | - | - | - | - | - | - |
| HUM30 | 0.71 | - | - | - | - | - | - | - | - | - | - | - | - |
| HUM31 | 0.69 | - | - | - | - | - | - | - | - | - | - | - | 0.28 |
| HUM32 | 0.65 | - | - | - | - | - | - | - | - | - | - | - | 0.24 |
| Pain1 | - | - | - | 0.74 | - | - | - | - | - | - | - | - | - |
| Pain2 | - | - | - | 0.74 | - | - | - | - | - | - | - | - | - |
| Pain3 | - | - | - | 0.77 | - | - | - | - | - | - | - | - | - |
| Pain4 | - | - | - | 0.72 | - | - | - | - | - | - | - | - | - |
| Pain5 | - | - | - | 0.72 | - | - | - | - | - | - | - | - | - |
| Pain6 | - | - | - | 0.57 | - | - | - | - | - | - | - | - | - |
| Pain7 | - | - | - | 0.7 | - | - | - | - | - | - | - | - | - |
| Pain8 | - | - | - | 0.6 | - | - | - | - | - | - | - | - | - |
| Pain9 | - | - | - | 0.69 | - | - | - | - | - | - | - | - | - |
| Pain10 | - | - | - | 0.64 | - | - | - | - | - | - | - | - | - |
| Pain11 | - | - | - | 0.62 | - | - | - | - | - | - | - | - | - |
| Pain12 | - | - | - | 0.66 | - | - | - | - | - | - | - | - | - |
| Pain13 | - | - | - | 0.64 | - | - | - | - | - | - | - | - | - |
| Anger1 | - | - | 0.67 | - | - | - | - | - | - | - | - | - | - |
| Anger2 | - | - | 0.64 | - | - | - | - | - | - | - | - | - | - |
| Anger3 | 0.22 | - | 0.38 | - | - | - | - | - | - | - | - | - | - |
| Anger4 | - | - | 0.65 | - | - | - | - | - | - | - | - | - | - |
| Anger5 | - | - | 0.74 | - | - | - | - | - | - | - | - | - | - |
| Anger6 | - | - | 0.53 | - | - | - | - | - | - | - | - | - | - |
| Anger7 | - | - | 0.56 | - | - | - | - | - | - | - | - | - | - |
| Anger8 | - | - | 0.59 | - | - | - | - | - | - | - | - | - | - |
| Anger9 | - | - | 0.58 | - | - | - | - | - | - | - | - | - | - |
| Anger10 | - | - | 0.56 | - | - | - | - | - | - | - | - | - | - |
| Anger11 | - | - | 0.52 | - | - | - | - | - | - | - | - | - | - |
| Anger12 | - | - | 0.59 | - | - | - | - | - | - | - | - | - | - |
| Anger13 | - | - | 0.59 | - | - | - | - | - | - | - | - | - | - |
| Anger14 | - | - | 0.63 | - | - | - | - | - | - | - | - | - | - |
| Anger15 | - | - | 0.72 | - | - | - | - | - | - | - | - | - | - |
| Anger16 | - | - | 0.71 | - | - | - | - | - | - | - | - | - | - |
| Anger17 | - | - | 0.76 | - | - | - | - | - | - | - | - | - | - |
| Anger18 | - | - | 0.71 | - | - | - | - | - | - | - | - | - | - |
| Anger19 | - | - | 0.58 | - | - | - | - | - | - | - | - | - | - |
| Anger20 | - | - | 0.51 | - | - | - | - | - | - | - | - | - | - |
| Anger21 | - | - | 0.69 | - | - | - | - | - | - | - | - | - | - |
| Anger22 | - | - | 0.64 | - | - | - | - | - | - | - | - | - | - |
| Agg1 | - | - | 0.22 | - | - | - | - | - | - | - | - | - | - |
| Agg2 | - | - | - | - | 0.27 | - | - | - | - | - | - | - | - |
| Agg3 | - | - | - | - | 0.26 | - | - | - | - | - | - | - | - |
| Agg4 | - | - | - | - | 0.27 | - | - | - | - | - | - | - | - |
| Stress1 | - | - | 0.25 | - | 0.23 | - | - | - | - | - | - | - | - |
| Stress2 | - | - | 0.21 | - | - | - | 0.22 | - | - | - | - | - | - |
| Stress3 | - | - | 0.26 | - | - | - | - | - | - | - | - | - | - |
| Stress4. | - | - | - | - | - | - | - | - | - | - | - | 0.59 | - |
| Stress5. | - | - | - | - | - | - | - | - | - | - | - | 0.59 | - |
| Stress6 | - | - | - | - | - | - | - | - | - | - | - | - | - |
| Stress7. | - | - | - | - | - | - | - | - | - | - | - | 0.7 | - |
| Stress8. | - | - | - | - | - | - | - | - | - | - | - | 0.56 | - |
| Stress9 | - | - | 0.47 | - | - | - | - | - | - | - | - | - | - |
| Stress10 | - | - | 0.24 | - | - | - | 0.2 | - | - | - | - | - | - |
| Impulse1 | - | - | - | - | 0.62 | - | - | - | - | - | - | - | - |
| Impulse2 | - | - | - | - | 0.63 | - | - | - | - | - | - | - | 0.2 |
| Impulse3 | - | - | - | - | 0.72 | - | - | - | - | - | - | - | - |
| Impulse4 | - | - | - | - | 0.77 | - | - | - | - | - | - | - | - |
| Impulse5 | - | - | - | - | 0.76 | - | - | - | - | - | - | - | - |
| Impulse6 | - | - | - | - | 0.7 | - | - | - | - | - | - | - | - |
| Impulse7 | - | - | - | - | 0.67 | - | - | - | - | - | - | - | - |
| Impulse8 | - | - | - | - | 0.65 | - | - | - | - | - | - | - | - |
| Impulse9 | - | - | - | - | 0.69 | - | - | - | - | - | - | - | - |
| Impulse10 | - | - | - | - | 0.68 | - | - | - | - | - | - | - | - |
| Impulse11. | - | - | 0.22 | - | - | - | - | - | - | - | 0.2 | 0.33 | - |
| Impulse12 | - | - | - | - | 0.68 | - | - | - | - | - | - | - | - |
| DT1 | - | - | - | - | - | - | - | - | 0.43 | - | - | - | - |
| DT2 | - | - | - | - | - | - | - | - | 0.42 | - | - | - | - |
| DT3 | - | - | - | - | - | - | - | - | 0.46 | - | - | - | - |
| DT4 | - | - | - | - | - | - | - | - | 0.43 | - | - | - | - |
| DT5 | - | - | - | - | - | - | - | - | 0.51 | - | - | - | - |
| DT6. | - | - | - | - | - | - | - | - | - | 0.22 | - | 0.34 | - |
| DT7 | - | - | - | - | - | - | - | - | 0.58 | - | - | - | - |
| DT8 | - | - | - | - | - | - | - | - | 0.74 | - | - | - | - |
| DT9 | - | - | - | - | - | - | - | - | 0.49 | - | - | - | - |
| DT10 | - | - | - | - | - | - | - | - | 0.55 | - | - | - | - |
| DT11 | - | - | - | - | - | - | - | - | 0.49 | - | - | - | - |
| DT12 | - | - | - | - | - | - | - | - | 0.48 | - | - | - | - |
| DT13 | - | - | - | - | - | - | - | - | 0.69 | - | - | - | - |
| DT14 | - | - | - | - | - | - | - | - | 0.75 | - | - | - | - |
| DT15 | - | - | - | - | - | - | - | - | 0.48 | - | - | - | - |
| BH.N1 | - | - | - | - | - | - | 0.27 | - | - | - | 0.21 | - | - |
| BH.N2 | - | - | - | - | - | - | 0.22 | 0.22 | - | - | 0.26 | - | - |
| BH.N3 | - | - | - | - | - | - | 0.25 | - | - | - | 0.22 | - | - |
| BH.P1. | - | - | - | - | - | - | - | - | - | - | 0.54 | 0.22 | - |
| BH.P2. | - | - | - | - | - | - | - | - | - | - | 0.56 | 0.2 | - |
| BH.P3. | - | - | - | - | - | - | - | - | - | - | 0.53 | - | - |

Note: Item abbreviations followed by . denote reverse coded items; - = loadings < .20; INQ = Interpersonal Needs Questionnaire, UCLA = UCLA Loneliness, STHS = State-Trait Hopelessness Scale, TBS = Thwarted Belongingness Scale, PB.VAS = Perceived Burdensomeness Visual Analog Scale Item (Bryan, 2019), DS = Defeat Scale, TRAP = Entrapment Scale, HUM = Humiliation, Pain = Psychological Pain, Agg = Aggression, Stress = Perceived Stress, Impulse = Negative Urgency, DT = Distress Tolerance, BH.N = Brief Hopelessness Negative, BH.P = Brief Hopelessness Positive.

**Table S2**. Factor Loading Patterns for 12-dimensional EIFA in Full Initial Item Pool.

|  | F1 | F2 | F3 | F4 | F5 | F6 | F7 | F8 | F9 | F10 | F11 | F12 |
| --- | --- | --- | --- | --- | --- | --- | --- | --- | --- | --- | --- | --- |
| INQ1 | - | - | - | - | - | - | - | - | - | - | 0.4 | - |
| INQ2 | - | - | - | - | - | - | - | - | - | - | 0.4 | - |
| INQ3 | - | - | - | - | - | - | - | - | - | - | 0.37 | - |
| INQ4 | - | - | - | - | - | - | - | - | - | - | 0.47 | - |
| INQ5 | - | - | - | - | - | - | - | - | - | - | 0.39 | - |
| INQ6 | - | - | - | 0.23 | - | - | - | - | - | - | 0.33 | - |
| INQ7. | - | - | - | - | - | - | - | - | - | 0.74 | - | - |
| INQ8. | - | - | - | - | - | - | - | - | - | 0.69 | - | - |
| INQ9. | - | - | - | - | - | - | - | - | - | 0.83 | - | - |
| INQ10. | - | - | - | - | - | - | - | - | - | 0.8 | - | - |
| INQ11 | - | 0.33 | - | - | - | - | - | - | - | - | - | - |
| INQ12 | - | 0.3 | - | - | - | - | - | - | - | - | - | - |
| INQ13. | - | - | - | - | - | - | - | - | - | 0.83 | - | - |
| INQ14. | - | - | - | - | - | - | - | - | - | 0.8 | - | - |
| INQ15. | - | - | - | - | - | - | - | - | - | 0.76 | - | - |
| UCLA1 | - | 0.64 | - | - | - | - | - | - | - | - | - | - |
| UCLA2 | - | 0.73 | - | - | - | - | - | - | - | - | - | - |
| UCLA3 | - | 0.63 | - | - | - | - | - | - | - | - | - | - |
| UCLA4 | - | 0.84 | - | - | - | - | - | - | - | - | - | - |
| UCLA5 | - | 0.69 | - | - | - | - | - | - | - | - | - | - |
| UCLA6 | - | 0.66 | - | - | - | - | - | - | - | - | - | - |
| UCLA7 | - | 0.73 | - | - | - | - | - | - | - | - | - | - |
| UCLA8 | - | 0.72 | - | - | - | - | - | - | - | - | - | - |
| UCLA9 | - | 0.68 | - | - | - | - | - | - | - | - | - | - |
| UCLA10 | - | 0.7 | - | - | - | - | - | - | - | - | - | - |
| UCLA11 | - | 0.7 | - | - | - | - | - | - | - | - | - | - |
| UCLA12 | - | 0.63 | - | - | - | - | - | - | - | - | - | - |
| UCLA13 | - | 0.61 | - | - | - | - | - | - | - | - | - | - |
| UCLA14 | - | 0.67 | - | - | - | - | - | - | - | - | - | - |
| UCLA15 | - | 0.72 | - | - | - | - | - | - | - | - | - | - |
| UCLA16 | - | 0.74 | - | - | - | - | - | - | - | - | - | - |
| UCLA17 | - | 0.57 | - | - | - | - | - | - | - | - | - | - |
| UCLA18 | - | 0.61 | - | - | - | - | - | - | - | - | - | - |
| UCLA19 | - | 0.64 | - | - | - | - | - | - | - | - | - | - |
| UCLA20 | - | 0.6 | - | - | - | - | - | - | - | - | - | - |
| STHS1 | - | - | - | - | - | 0.24 | - | - | - | - | - | - |
| STHS2 | - | - | - | - | - | 0.26 | - | - | - | - | 0.24 | - |
| STHS3. | - | - | - | - | - | - | - | - | 0.7 | - | - | - |
| STHS4 | - | - | - | - | - | 0.24 | - | - | - | - | 0.21 | - |
| STHS5 | - | - | - | - | - | 0.24 | - | - | - | - | - | - |
| STHS6. | - | - | - | - | - | - | - | - | 0.72 | - | - | - |
| STHS7. | - | - | - | - | - | - | - | - | 0.77 | - | - | - |
| STHS8 | - | - | - | - | - | 0.21 | - | - | - | - | 0.26 | - |
| STHS9. | - | - | - | - | - | - | - | - | 0.75 | - | - | - |
| STHS10 | - | - | - | - | - | 0.29 | - | - | - | - | - | - |
| TBS1 | - | 0.34 | - | - | - | - | - | - | - | - | - | - |
| TBS2 | - | 0.3 | - | - | - | - | - | - | - | - | - | - |
| TBS3 | - | 0.25 | - | - | - | - | - | - | - | 0.23 | - | - |
| TBS4 | - | 0.33 | - | - | - | - | - | - | - | 0.24 | - | - |
| TBS5 | - | 0.27 | - | - | - | - | - | - | - | - | - | - |
| TBS6 | - | 0.34 | - | - | - | - | - | - | - | - | - | - |
| TBS7 | - | 0.37 | - | - | - | - | - | - | - | 0.21 | - | - |
| TBS8 | - | 0.38 | - | - | - | - | - | - | - | 0.22 | - | - |
| PB.VAS | - | - | - | - | - | - | - | - | - | - | - | - |
| DS1 | - | - | - | - | - | 0.52 | - | - | - | - | - | - |
| DS2. | - | - | - | - | - | 0.21 | - | - | 0.44 | - | - | - |
| DS3 | - | - | - | - | - | 0.54 | - | - | - | - | - | - |
| DS4. | - | - | - | - | - | 0.27 | - | - | 0.42 | - | - | - |
| DS5 | - | - | - | - | - | 0.49 | - | - | - | - | - | - |
| DS6 | - | - | - | - | - | 0.5 | - | - | - | - | - | - |
| DS7 | - | - | - | - | - | 0.54 | - | - | - | - | - | - |
| DS8 | - | - | - | - | - | 0.49 | - | - | - | - | - | - |
| DS9. | - | - | - | - | - | - | - | - | 0.5 | 0.21 | - | - |
| DS10 | - | - | - | - | - | 0.52 | - | - | - | - | - | - |
| DS11 | - | - | - | - | - | 0.5 | - | - | - | - | - | - |
| DS12 | - | - | - | - | - | 0.52 | - | - | - | - | - | - |
| DS13 | - | - | - | - | - | 0.45 | - | - | - | - | - | - |
| DS14 | - | - | - | - | - | 0.49 | - | - | - | - | - | - |
| DS15 | - | - | - | - | - | 0.51 | - | - | - | - | - | - |
| DS16 | - | - | - | - | - | 0.44 | - | - | - | - | - | - |
| TRAP1 | - | - | - | - | - | - | - | 0.38 | - | - | - | - |
| TRAP2 | - | - | - | - | - | - | - | 0.38 | - | - | - | - |
| TRAP3 | - | - | - | 0.21 | - | - | - | 0.46 | - | - | - | - |
| TRAP4 | - | - | - | 0.25 | - | - | - | 0.42 | - | - | - | - |
| TRAP5 | - | - | - | - | - | - | - | 0.48 | - | - | - | - |
| TRAP6 | - | - | - | - | - | - | - | 0.42 | - | - | - | - |
| TRAP7 | - | - | - | 0.21 | - | - | - | 0.44 | - | - | - | - |
| TRAP8 | - | - | - | - | - | - | - | 0.5 | - | - | - | - |
| TRAP9 | - | - | - | - | - | - | - | 0.45 | - | - | - | - |
| TRAP10 | - | - | - | - | - | - | - | 0.49 | - | - | - | - |
| TRAP11 | - | - | - | - | - | - | - | 0.46 | - | - | - | - |
| TRAP12 | - | - | - | - | - | - | - | 0.48 | - | - | - | - |
| TRAP13 | - | - | - | - | - | - | - | 0.47 | - | - | - | - |
| TRAP14 | - | - | - | - | - | - | - | 0.41 | - | - | - | - |
| TRAP15 | - | - | - | - | - | - | - | 0.49 | - | - | - | - |
| TRAP16 | - | - | - | - | - | - | - | 0.45 | - | - | - | - |
| HUM1 | 0.61 | - | - | - | - | - | - | - | - | - | - | - |
| HUM2 | 0.69 | - | - | - | - | - | - | - | - | - | - | - |
| HUM3 | 0.73 | - | - | - | - | - | - | - | - | - | - | - |
| HUM4 | 0.69 | - | - | - | - | - | - | - | - | - | - | - |
| HUM5 | 0.76 | - | - | - | - | - | - | - | - | - | - | - |
| HUM6 | 0.78 | - | - | - | - | - | - | - | - | - | - | - |
| HUM7 | 0.8 | - | - | - | - | - | - | - | - | - | - | - |
| HUM8 | 0.79 | - | - | - | - | - | - | - | - | - | - | - |
| HUM9 | 0.8 | - | - | - | - | - | - | - | - | - | - | - |
| HUM10 | 0.82 | - | - | - | - | - | - | - | - | - | - | - |
| HUM11 | 0.86 | - | - | - | - | - | - | - | - | - | - | - |
| HUM12 | 0.81 | - | - | - | - | - | - | - | - | - | - | - |
| HUM13 | 0.82 | - | - | - | - | - | - | - | - | - | - | - |
| HUM14 | 0.87 | - | - | - | - | - | - | - | - | - | - | - |
| HUM15 | 0.89 | - | - | - | - | - | - | - | - | - | - | - |
| HUM16 | 0.8 | - | - | - | - | - | - | - | - | - | - | - |
| HUM17 | 0.91 | - | - | - | - | - | - | - | - | - | - | - |
| HUM18 | 0.84 | - | - | - | - | - | - | - | - | - | - | - |
| HUM19 | 0.82 | - | - | - | - | - | - | - | - | - | - | - |
| HUM20 | 0.86 | - | - | - | - | - | - | - | - | - | - | - |
| HUM21 | 0.88 | - | - | - | - | - | - | - | - | - | - | - |
| HUM22 | 0.84 | - | - | - | - | - | - | - | - | - | - | - |
| HUM23 | 0.79 | - | - | - | - | - | - | - | - | - | - | - |
| HUM24 | 0.85 | - | - | - | - | - | - | - | - | - | - | - |
| HUM25 | 0.84 | - | - | - | - | - | - | - | - | - | - | - |
| HUM26 | 0.77 | - | - | - | - | - | - | - | - | - | - | - |
| HUM27 | 0.76 | - | - | - | - | - | - | - | - | - | - | - |
| HUM28 | 0.75 | - | - | - | - | - | - | - | - | - | - | - |
| HUM29 | 0.81 | - | - | - | - | - | - | - | - | - | - | - |
| HUM30 | 0.73 | - | - | - | - | - | - | - | - | - | - | - |
| HUM31 | 0.72 | - | - | - | - | - | - | - | - | - | - | 0.24 |
| HUM32 | 0.68 | - | - | - | - | - | - | - | - | - | - | 0.21 |
| Pain1 | - | - | - | 0.76 | - | - | - | - | - | - | - | - |
| Pain2 | - | - | - | 0.75 | - | - | - | - | - | - | - | - |
| Pain3 | - | - | - | 0.79 | - | - | - | - | - | - | - | - |
| Pain4 | - | - | - | 0.74 | - | - | - | - | - | - | - | - |
| Pain5 | - | - | - | 0.75 | - | - | - | - | - | - | - | - |
| Pain6 | - | - | - | 0.58 | - | - | - | - | - | - | - | - |
| Pain7 | - | - | - | 0.71 | - | - | - | - | - | - | - | - |
| Pain8 | - | - | - | 0.61 | - | - | - | - | - | - | - | - |
| Pain9 | - | - | - | 0.7 | - | - | - | - | - | - | - | - |
| Pain10 | - | - | - | 0.67 | - | - | - | - | - | - | - | - |
| Pain11 | - | - | - | 0.65 | - | - | - | - | - | - | - | - |
| Pain12 | - | - | - | 0.68 | - | - | - | - | - | - | - | - |
| Pain13 | - | - | - | 0.66 | - | - | - | - | - | - | - | - |
| Anger1 | - | - | 0.69 | - | - | - | - | - | - | - | - | - |
| Anger2 | - | - | 0.67 | - | - | - | - | - | - | - | - | - |
| Anger3 | 0.23 | - | 0.39 | - | - | - | - | - | - | - | - | - |
| Anger4 | - | - | 0.67 | - | - | - | - | - | - | - | - | - |
| Anger5 | - | - | 0.77 | - | - | - | - | - | - | - | - | - |
| Anger6 | - | - | 0.55 | - | - | - | - | - | - | - | - | - |
| Anger7 | - | - | 0.58 | - | - | - | - | - | - | - | - | - |
| Anger8 | - | - | 0.61 | - | - | - | - | - | - | - | - | - |
| Anger9 | - | - | 0.6 | - | - | - | - | - | - | - | - | - |
| Anger10 | - | - | 0.57 | - | - | - | - | - | - | - | - | - |
| Anger11 | - | - | 0.53 | - | - | - | - | - | - | - | - | - |
| Anger12 | - | - | 0.61 | - | - | - | - | - | - | - | - | - |
| Anger13 | - | - | 0.61 | - | - | - | - | - | - | - | - | - |
| Anger14 | - | - | 0.66 | - | - | - | - | - | - | - | - | - |
| Anger15 | - | - | 0.74 | - | - | - | - | - | - | - | - | - |
| Anger16 | - | - | 0.74 | - | - | - | - | - | - | - | - | - |
| Anger17 | - | - | 0.79 | - | - | - | - | - | - | - | - | - |
| Anger18 | - | - | 0.74 | - | - | - | - | - | - | - | - | - |
| Anger19 | - | - | 0.6 | - | - | - | - | - | - | - | - | - |
| Anger20 | - | - | 0.53 | - | - | - | - | - | - | - | - | - |
| Anger21 | - | - | 0.72 | - | - | - | - | - | - | - | - | - |
| Anger22 | - | - | 0.66 | - | - | - | - | - | - | - | - | - |
| Agg1 | - | - | 0.24 | - | - | - | - | - | - | - | - | - |
| Agg2 | - | - | - | - | 0.28 | - | - | - | - | - | 0.26 | - |
| Agg3 | - | - | - | - | 0.27 | - | - | - | - | - | 0.3 | - |
| Agg4 | - | - | - | - | 0.28 | - | - | - | - | - | 0.26 | - |
| Stress1 | - | - | 0.26 | - | 0.23 | - | - | - | - | - | - | - |
| Stress2 | - | - | 0.22 | - | - | 0.23 | - | - | - | - | - | - |
| Stress3 | - | - | 0.27 | - | - | - | - | - | - | - | - | - |
| Stress4. | - | - | - | - | - | - | - | - | 0.53 | - | - | - |
| Stress5. | - | - | - | - | - | - | - | - | 0.54 | - | - | - |
| Stress6 | - | - | 0.2 | - | - | - | - | - | - | - | - | - |
| Stress7. | - | - | - | - | - | - | - | - | 0.53 | - | - | - |
| Stress8. | - | - | - | - | - | - | - | - | 0.56 | - | - | - |
| Stress9 | - | - | 0.48 | - | - | - | - | - | - | - | - | - |
| Stress10 | - | - | 0.26 | - | - | 0.21 | - | - | - | - | - | - |
| Impulse1 | - | - | - | - | 0.63 | - | - | - | - | - | - | - |
| Impulse2 | - | - | - | - | 0.64 | - | - | - | - | - | - | - |
| Impulse3 | - | - | - | - | 0.73 | - | - | - | - | - | - | - |
| Impulse4 | - | - | - | - | 0.79 | - | - | - | - | - | - | - |
| Impulse5 | - | - | - | - | 0.77 | - | - | - | - | - | - | - |
| Impulse6 | - | - | - | - | 0.71 | - | - | - | - | - | - | - |
| Impulse7 | - | - | - | - | 0.68 | - | - | - | - | - | - | - |
| Impulse8 | - | - | - | - | 0.66 | - | - | - | - | - | - | - |
| Impulse9 | - | - | - | - | 0.7 | - | - | - | - | - | - | - |
| Impulse10 | - | - | - | - | 0.69 | - | - | - | - | - | - | - |
| Impulse11. | - | - | 0.23 | - | - | - | - | - | 0.42 | - | - | - |
| Impulse12 | - | - | - | - | 0.69 | - | - | - | - | - | - | - |
| DT1 | - | - | - | - | - | - | 0.43 | - | - | - | - | - |
| DT2 | - | - | - | 0.21 | - | - | 0.42 | - | - | - | - | - |
| DT3 | - | - | - | - | - | - | 0.47 | - | - | - | - | - |
| DT4 | - | - | - | - | - | - | 0.44 | - | - | - | - | - |
| DT5 | - | - | - | - | - | - | 0.51 | - | - | - | - | - |
| DT6. | - | - | - | - | - | - | - | - | 0.27 | 0.24 | - | - |
| DT7 | - | - | - | - | - | - | 0.58 | - | - | - | - | - |
| DT8 | - | - | - | - | - | - | 0.75 | - | - | - | - | - |
| DT9 | - | - | - | - | - | - | 0.5 | - | - | - | - | - |
| DT10 | - | - | - | - | - | - | 0.55 | - | - | - | - | - |
| DT11 | - | - | - | - | - | - | 0.5 | - | - | - | - | - |
| DT12 | - | - | - | - | - | - | 0.49 | - | - | - | - | - |
| DT13 | - | - | - | - | - | - | 0.7 | - | - | - | - | - |
| DT14 | - | - | - | - | - | - | 0.75 | - | - | - | - | - |
| DT15 | - | - | - | - | - | - | 0.49 | - | - | - | - | - |
| BH.N1 | - | - | - | - | - | 0.3 | - | - | - | - | - | - |
| BH.N2 | - | - | - | - | - | 0.24 | - | - | - | - | 0.22 | - |
| BH.N3 | - | - | - | - | - | 0.27 | - | - | - | - | - | - |
| BH.P1. | - | - | - | - | - | - | - | - | 0.69 | - | - | - |
| BH.P2. | - | - | - | - | - | - | - | - | 0.69 | - | - | - |
| BH.P3. | - | - | - | - | - | - | - | - | 0.66 | - | - | - |

Note: Item abbreviations followed by . denote reverse coded items; - = loadings < .20; INQ = Interpersonal Needs Questionnaire, UCLA = UCLA Loneliness, STHS = State-Trait Hopelessness Scale, TBS = Thwarted Belongingness Scale, PB.VAS = Perceived Burdensomeness Visual Analog Scale Item (Bryan, 2019), DS = Defeat Scale, TRAP = Entrapment Scale, HUM = Humiliation, Pain = Psychological Pain, Agg = Aggression, Stress = Perceived Stress, Impulse = Negative Urgency, DT = Distress Tolerance, BH.N = Brief Hopelessness Negative, BH.P = Brief Hopelessness Positive.

**Table S3**. Factor Loading Patterns for 12-dimensional EIFA in Reduced Item Pool.

|  | F1 | F2 | F3 | F4 | F5 | F6 | F7 | F8 | F9 | F10 | F11 | F12 |
| --- | --- | --- | --- | --- | --- | --- | --- | --- | --- | --- | --- | --- |
| INQ1 | - | - | - | - | - | - | - | - | 0.53 | - | - | - |
| INQ2 | - | - | - | - | - | - | - | - | 0.51 | - | - | - |
| INQ3 | - | - | - | - | - | - | - | - | 0.48 | - | - | - |
| INQ4 | - | - | - | - | - | - | - | - | 0.58 | - | - | - |
| INQ5 | - | - | - | - | - | - | - | - | 0.49 | - | - | - |
| INQ6 | - | - | - | - | - | - | - | - | 0.45 | - | - | - |
| INQ7. | - | - | - | - | - | - | - | - | - | 0.74 | - | - |
| INQ8. | - | - | - | - | - | - | - | - | - | 0.69 | - | - |
| INQ9. | - | - | - | - | - | - | - | - | - | 0.82 | - | - |
| INQ10. | - | - | - | - | - | - | - | - | - | 0.78 | - | - |
| INQ11 | - | 0.31 | - | - | - | - | - | - | 0.22 | - | - | - |
| INQ12 | - | 0.28 | - | - | - | - | - | - | 0.23 | - | - | - |
| INQ13. | - | - | - | - | - | - | - | - | - | 0.82 | - | - |
| INQ14. | - | - | - | - | - | - | - | - | - | 0.79 | - | - |
| INQ15. | - | - | - | - | - | - | - | - | - | 0.76 | - | - |
| UCLA1 | - | 0.64 | - | - | - | - | - | - | - | - | - | - |
| UCLA2 | - | 0.72 | - | - | - | - | - | - | - | - | - | - |
| UCLA3 | - | 0.61 | - | - | - | - | - | - | - | - | - | - |
| UCLA4 | - | 0.83 | - | - | - | - | - | - | - | - | - | - |
| UCLA5 | - | 0.67 | - | - | - | - | - | - | - | - | - | - |
| UCLA6 | - | 0.64 | - | - | - | - | - | - | - | - | - | - |
| UCLA7 | - | 0.72 | - | - | - | - | - | - | - | - | - | - |
| UCLA8 | - | 0.71 | - | - | - | - | - | - | - | - | - | - |
| UCLA9 | - | 0.67 | - | - | - | - | - | - | - | - | - | - |
| UCLA10 | - | 0.69 | - | - | - | - | - | - | - | - | - | - |
| UCLA11 | - | 0.69 | - | - | - | - | - | - | - | - | - | - |
| UCLA12 | - | 0.63 | - | - | - | - | - | - | - | - | - | - |
| UCLA13 | - | 0.61 | - | - | - | - | - | - | - | - | - | - |
| UCLA14 | - | 0.65 | - | - | - | - | - | - | - | - | - | - |
| UCLA15 | - | 0.71 | - | - | - | - | - | - | - | - | - | - |
| UCLA16 | - | 0.72 | - | - | - | - | - | - | - | - | - | - |
| UCLA17 | - | 0.55 | - | - | - | - | - | - | - | - | - | - |
| UCLA18 | - | 0.6 | - | - | - | - | - | - | - | - | - | - |
| UCLA19 | - | 0.62 | - | - | - | - | - | - | - | - | - | - |
| UCLA20 | - | 0.59 | - | - | - | - | - | - | - | - | - | - |
| STHS1 | - | - | - | - | - | 0.24 | - | - | 0.26 | - | - | - |
| STHS2 | - | - | - | - | - | 0.27 | - | - | 0.28 | - | - | - |
| STHS3. | - | - | - | - | - | - | - | - | - | - | 0.72 | - |
| STHS4 | - | - | - | - | - | 0.25 | - | - | 0.28 | - | - | - |
| STHS5 | - | - | - | - | - | 0.24 | - | - | 0.24 | - | - | - |
| STHS6. | - | - | - | - | - | - | - | - | - | - | 0.73 | - |
| STHS7. | - | - | - | - | - | - | - | - | - | - | 0.8 | - |
| STHS8 | - | - | - | - | - | 0.21 | - | - | 0.32 | - | - | - |
| STHS9. | - | - | - | - | - | - | - | - | - | - | 0.76 | - |
| STHS10 | - | - | - | - | - | 0.29 | - | - | - | - | - | - |
| TBS1 | - | 0.33 | - | - | - | - | - | - | - | - | - | - |
| TBS2 | - | 0.29 | - | - | - | - | - | - | - | - | - | - |
| TBS3 | - | 0.24 | - | - | - | - | - | - | 0.2 | 0.22 | - | - |
| TBS4 | - | 0.32 | - | - | - | - | - | - | - | 0.22 | - | - |
| TBS5 | - | 0.26 | - | - | - | - | - | - | - | - | - | - |
| TBS6 | - | 0.32 | - | - | - | - | - | - | - | - | - | - |
| TBS7 | - | 0.35 | - | - | - | - | - | - | 0.22 | - | - | - |
| TBS8 | - | 0.37 | - | - | - | - | - | - | - | 0.21 | - | - |
| PB.VAS | - | - | - | - | - | - | - | - | - | - | - | - |
| DS1 | - | - | - | - | - | 0.53 | - | - | - | - | - | - |
| DS2. | - | - | - | - | - | 0.22 | - | - | - | - | 0.45 | - |
| DS3 | - | - | - | - | - | 0.54 | - | - | - | - | - | - |
| DS4. | - | - | - | - | - | 0.28 | - | - | - | - | 0.44 | - |
| DS5 | - | - | - | - | - | 0.5 | - | - | - | - | - | - |
| DS6 | - | - | - | - | - | 0.5 | - | - | - | - | - | - |
| DS7 | - | - | - | - | - | 0.55 | - | - | - | - | - | - |
| DS8 | - | - | - | - | - | 0.5 | - | - | - | - | - | - |
| DS9. | - | - | - | - | - | - | - | - | - | 0.24 | 0.48 | - |
| DS10 | - | - | - | - | - | 0.53 | - | - | - | - | - | - |
| DS11 | - | - | - | - | - | 0.5 | - | - | - | - | - | - |
| DS12 | - | - | - | - | - | 0.54 | - | - | - | - | - | - |
| DS13 | - | - | - | - | - | 0.46 | - | - | - | - | - | - |
| DS14 | - | - | - | - | - | 0.49 | - | - | - | - | - | - |
| DS15 | - | - | - | - | - | 0.52 | - | - | - | - | - | - |
| DS16 | - | - | - | - | - | 0.45 | - | - | - | - | - | - |
| TRAP1 | - | - | - | - | - | - | 0.39 | - | - | - | - | - |
| TRAP2 | - | - | - | - | - | - | 0.39 | - | - | - | - | - |
| TRAP3 | - | - | - | 0.21 | - | - | 0.47 | - | - | - | - | - |
| TRAP4 | - | - | - | 0.24 | - | - | 0.44 | - | - | - | - | - |
| TRAP5 | - | - | - | - | - | - | 0.49 | - | - | - | - | - |
| TRAP6 | - | - | - | - | - | - | 0.43 | - | - | - | - | - |
| TRAP7 | - | - | - | 0.2 | - | - | 0.47 | - | - | - | - | - |
| TRAP8 | - | - | - | - | - | - | 0.52 | - | - | - | - | - |
| TRAP9 | - | - | - | - | - | - | 0.48 | - | - | - | - | - |
| TRAP10 | - | - | - | - | - | - | 0.51 | - | - | - | - | - |
| TRAP11 | - | - | - | - | - | - | 0.49 | - | - | - | - | - |
| TRAP12 | - | - | - | - | - | - | 0.52 | - | - | - | - | - |
| TRAP13 | - | - | - | - | - | - | 0.5 | - | - | - | - | - |
| TRAP14 | - | - | - | - | - | - | 0.42 | - | - | - | - | - |
| TRAP15 | - | - | - | - | - | - | 0.51 | - | - | - | - | - |
| TRAP16 | - | - | - | - | - | - | 0.48 | - | - | - | - | - |
| HUM1 | 0.61 | - | - | - | - | - | - | - | - | - | - | 0.22 |
| HUM2 | 0.68 | - | - | - | - | - | - | - | - | - | - | 0.23 |
| HUM3 | 0.72 | - | - | - | - | - | - | - | - | - | - | - |
| HUM4 | 0.68 | - | - | - | - | - | - | - | - | - | - | - |
| HUM5 | 0.75 | - | - | - | - | - | - | - | - | - | - | - |
| HUM6 | 0.77 | - | - | - | - | - | - | - | - | - | - | - |
| HUM7 | 0.79 | - | - | - | - | - | - | - | - | - | - | - |
| HUM8 | 0.78 | - | - | - | - | - | - | - | - | - | - | - |
| HUM9 | 0.79 | - | - | - | - | - | - | - | - | - | - | - |
| HUM10 | 0.8 | - | - | - | - | - | - | - | - | - | - | - |
| HUM11 | 0.84 | - | - | - | - | - | - | - | - | - | - | - |
| HUM12 | 0.8 | - | - | - | - | - | - | - | - | - | - | - |
| HUM13 | 0.8 | - | - | - | - | - | - | - | - | - | - | - |
| HUM14 | 0.86 | - | - | - | - | - | - | - | - | - | - | - |
| HUM15 | 0.87 | - | - | - | - | - | - | - | - | - | - | - |
| HUM16 | 0.78 | - | - | - | - | - | - | - | - | - | - | - |
| HUM17 | 0.89 | - | - | - | - | - | - | - | - | - | - | - |
| HUM18 | 0.82 | - | - | - | - | - | - | - | - | - | - | - |
| HUM19 | 0.8 | - | - | - | - | - | - | - | - | - | - | - |
| HUM20 | 0.85 | - | - | - | - | - | - | - | - | - | - | - |
| HUM21 | 0.86 | - | - | - | - | - | - | - | - | - | - | - |
| HUM22 | 0.83 | - | - | - | - | - | - | - | - | - | - | - |
| HUM23 | 0.78 | - | - | - | - | - | - | - | - | - | - | - |
| HUM24 | 0.84 | - | - | - | - | - | - | - | - | - | - | - |
| HUM25 | 0.82 | - | - | - | - | - | - | - | - | - | - | - |
| HUM26 | 0.76 | - | - | - | - | - | - | - | - | - | - | - |
| HUM27 | 0.75 | - | - | - | - | - | - | - | - | - | - | - |
| HUM28 | 0.74 | - | - | - | - | - | - | - | - | - | - | - |
| HUM29 | 0.8 | - | - | - | - | - | - | - | - | - | - | - |
| HUM30 | 0.71 | - | - | - | - | - | - | - | - | - | - | - |
| HUM31 | 0.7 | - | - | - | - | - | - | - | - | - | - | - |
| HUM32 | 0.66 | - | - | - | - | - | - | - | - | - | - | - |
| Pain1 | - | - | - | 0.74 | - | - | - | - | - | - | - | - |
| Pain2 | - | - | - | 0.74 | - | - | - | - | - | - | - | - |
| Pain3 | - | - | - | 0.78 | - | - | - | - | - | - | - | - |
| Pain4 | - | - | - | 0.72 | - | - | - | - | - | - | - | - |
| Pain5 | - | - | - | 0.73 | - | - | - | - | - | - | - | - |
| Pain6 | - | - | - | 0.57 | - | - | - | - | - | - | - | - |
| Pain7 | - | - | - | 0.7 | - | - | - | - | - | - | - | - |
| Pain8 | - | - | - | 0.6 | - | - | - | - | - | - | - | - |
| Pain9 | - | - | - | 0.69 | - | - | - | - | - | - | - | - |
| Pain10 | - | - | - | 0.65 | - | - | - | - | - | - | - | - |
| Pain11 | - | - | - | 0.63 | - | - | - | - | - | - | - | - |
| Pain12 | - | - | - | 0.66 | - | - | - | - | - | - | - | - |
| Pain13 | - | - | - | 0.64 | - | - | - | - | - | - | - | - |
| Anger1 | - | - | 0.69 | - | - | - | - | - | - | - | - | - |
| Anger2 | - | - | 0.67 | - | - | - | - | - | - | - | - | - |
| Anger3 | 0.22 | - | 0.4 | - | - | - | - | - | - | - | - | - |
| Anger4 | - | - | 0.66 | - | - | - | - | - | - | - | - | - |
| Anger5 | - | - | 0.76 | - | - | - | - | - | - | - | - | - |
| Anger6 | - | - | 0.56 | - | - | - | - | - | - | - | - | - |
| Anger7 | - | - | 0.59 | - | - | - | - | - | - | - | - | - |
| Anger8 | - | - | 0.61 | - | - | - | - | - | - | - | - | - |
| Anger9 | - | - | 0.61 | - | - | - | - | - | - | - | - | - |
| Anger10 | - | - | 0.58 | - | - | - | - | - | - | - | - | - |
| Anger11 | - | - | 0.54 | - | - | - | - | - | - | - | - | - |
| Anger12 | - | - | 0.61 | - | - | - | - | - | - | - | - | - |
| Anger13 | - | - | 0.61 | - | - | - | - | - | - | - | - | - |
| Anger14 | - | - | 0.66 | - | - | - | - | - | - | - | - | - |
| Anger15 | - | - | 0.74 | - | - | - | - | - | - | - | - | - |
| Anger16 | - | - | 0.75 | - | - | - | - | - | - | - | - | - |
| Anger17 | - | - | 0.78 | - | - | - | - | - | - | - | - | - |
| Anger18 | - | - | 0.74 | - | - | - | - | - | - | - | - | - |
| Anger19 | - | - | 0.6 | - | - | - | - | - | - | - | - | - |
| Anger20 | - | - | 0.53 | - | - | - | - | - | - | - | - | - |
| Anger21 | - | - | 0.72 | - | - | - | - | - | - | - | - | - |
| Anger22 | - | - | 0.66 | - | - | - | - | - | - | - | - | - |
| Agg1 | - | - | 0.23 | - | - | - | - | - | - | - | - | 0.22 |
| Agg2 | - | - | - | - | 0.28 | - | - | - | 0.21 | - | - | 0.28 |
| Agg3 | - | - | - | - | 0.27 | - | - | - | 0.24 | - | - | 0.33 |
| Agg4 | - | - | - | - | 0.28 | - | - | - | - | - | - | 0.3 |
| Impulse1 | - | - | - | - | 0.62 | - | - | - | - | - | - | - |
| Impulse2 | - | - | - | - | 0.63 | - | - | - | - | - | - | - |
| Impulse3 | - | - | - | - | 0.72 | - | - | - | - | - | - | - |
| Impulse4 | - | - | - | - | 0.77 | - | - | - | - | - | - | - |
| Impulse5 | - | - | - | - | 0.76 | - | - | - | - | - | - | - |
| Impulse6 | - | - | - | - | 0.7 | - | - | - | - | - | - | - |
| Impulse7 | - | - | - | - | 0.67 | - | - | - | - | - | - | - |
| Impulse8 | - | - | - | - | 0.64 | - | - | - | - | - | - | - |
| Impulse9 | - | - | - | - | 0.68 | - | - | - | - | - | - | - |
| Impulse10 | - | - | - | - | 0.67 | - | - | - | - | - | - | - |
| Impulse11. | - | - | 0.24 | - | - | - | - | - | - | 0.21 | 0.4 | - |
| Impulse12 | - | - | - | - | 0.68 | - | - | - | - | - | - | - |
| DT1 | - | - | - | - | - | - | - | 0.42 | - | - | - | - |
| DT2 | - | - | - | - | - | - | - | 0.42 | - | - | - | - |
| DT3 | - | - | - | - | - | - | - | 0.46 | - | - | - | - |
| DT4 | - | - | - | - | - | - | - | 0.43 | - | - | - | - |
| DT5 | - | - | - | - | - | - | - | 0.5 | - | - | - | - |
| DT6. | - | - | - | - | - | - | - | - | - | 0.28 | 0.25 | - |
| DT7 | - | - | - | - | - | - | - | 0.57 | - | - | - | - |
| DT8 | - | - | - | - | - | - | - | 0.73 | - | - | - | - |
| DT9 | - | - | - | - | - | - | - | 0.49 | - | - | - | - |
| DT10 | - | - | - | - | - | - | - | 0.54 | - | - | - | - |
| DT11 | - | - | - | - | - | - | - | 0.49 | - | - | - | - |
| DT12 | - | - | - | - | - | - | - | 0.48 | - | - | - | - |
| DT13 | - | - | - | - | - | - | - | 0.68 | - | - | - | - |
| DT14 | - | - | - | - | - | - | - | 0.74 | - | - | - | - |
| DT15 | - | - | - | - | - | - | - | 0.48 | - | - | - | - |
| BH.N1 | - | - | - | - | - | 0.29 | - | - | 0.2 | - | - | - |
| BH.N2 | - | - | - | - | - | 0.24 | - | - | 0.24 | - | - | - |
| BH.N3 | - | - | - | - | - | 0.27 | - | - | - | - | - | - |
| BH.P1. | - | - | - | - | - | - | - | - | - | - | 0.68 | - |
| BH.P2. | - | - | - | - | - | - | - | - | - | - | 0.68 | - |
| BH.P3. | - | - | - | - | - | - | - | - | - | - | 0.66 | - |

Note: Item abbreviations followed by . denote reverse coded items; - = loadings < .20; INQ = Interpersonal Needs Questionnaire, UCLA = UCLA Loneliness, STHS = State-Trait Hopelessness Scale, TBS = Thwarted Belongingness Scale, PB.VAS = Perceived Burdensomeness Visual Analog Scale Item (Bryan, 2019), DS = Defeat Scale, TRAP = Entrapment Scale, HUM = Humiliation, Pain = Psychological Pain, Agg = Aggression, Impulse = Negative Urgency, DT = Distress Tolerance, BH.N = Brief Hopelessness Negative, BH.P = Brief Hopelessness Positive.

**Table S4**. Factor Loading Patterns for 11-dimensional EIFA in Reduced Item Pool.

|  | F1 | F2 | F3 | F4 | F5 | F6 | F7 | F8 | F9 | F10 | F11 |
| --- | --- | --- | --- | --- | --- | --- | --- | --- | --- | --- | --- |
| INQ1 | - | 0.21 | 0.21 | - | - | 0.2 | - | - | - | - | 0.25 |
| INQ2 | - | 0.24 | - | - | - | - | - | - | - | - | 0.25 |
| INQ3 | - | - | 0.22 | - | - | - | - | - | - | - | 0.23 |
| INQ4 | - | - | 0.2 | - | - | - | - | - | - | - | 0.32 |
| INQ5 | - | 0.22 | - | - | - | - | - | - | - | - | 0.28 |
| INQ6 | - | - | 0.26 | - | - | - | - | - | - | - | - |
| INQ7. | - | - | - | - | - | - | - | - | 0.74 | - | - |
| INQ8. | - | - | - | - | - | - | - | - | 0.7 | - | - |
| INQ9. | - | - | - | - | - | - | - | - | 0.83 | - | - |
| INQ10. | - | - | - | - | - | - | - | - | 0.8 | - | - |
| INQ11 | - | 0.38 | - | - | - | - | - | - | - | - | - |
| INQ12 | - | 0.35 | - | - | - | - | - | - | - | - | - |
| INQ13. | - | - | - | - | - | - | - | - | 0.83 | - | - |
| INQ14. | - | - | - | - | - | - | - | - | 0.8 | - | - |
| INQ15. | - | - | - | - | - | - | - | - | 0.77 | - | - |
| UCLA1 | - | 0.6 | - | - | - | - | - | - | - | - | - |
| UCLA2 | - | 0.72 | - | - | - | - | - | - | - | - | - |
| UCLA3 | - | 0.57 | - | - | - | - | - | - | - | - | - |
| UCLA4 | - | 0.81 | - | - | - | - | - | - | - | - | - |
| UCLA5 | - | 0.67 | - | - | - | - | - | - | - | - | - |
| UCLA6 | - | 0.61 | - | - | - | - | - | - | - | - | - |
| UCLA7 | - | 0.72 | - | - | - | - | - | - | - | - | - |
| UCLA8 | - | 0.71 | - | - | - | - | - | - | - | - | - |
| UCLA9 | - | 0.67 | - | - | - | - | - | - | - | - | - |
| UCLA10 | - | 0.67 | - | - | - | - | - | - | - | - | - |
| UCLA11 | - | 0.68 | - | - | - | - | - | - | - | - | - |
| UCLA12 | - | 0.64 | - | - | - | - | - | - | - | - | - |
| UCLA13 | - | 0.61 | - | - | - | - | - | - | - | - | - |
| UCLA14 | - | 0.62 | - | - | - | - | - | - | - | - | - |
| UCLA15 | - | 0.7 | - | - | - | - | - | - | - | - | - |
| UCLA16 | - | 0.71 | - | - | - | - | - | - | - | - | - |
| UCLA17 | - | 0.53 | - | - | - | - | - | - | - | - | - |
| UCLA18 | - | 0.61 | - | - | - | - | - | - | - | - | - |
| UCLA19 | - | 0.62 | - | - | - | - | - | - | - | - | - |
| UCLA20 | - | 0.59 | - | - | - | - | - | - | - | - | - |
| STHS1 | - | - | - | - | - | - | 0.31 | - | - | - | - |
| STHS2 | - | - | - | - | - | - | 0.34 | - | - | - | - |
| STHS3. | - | - | - | - | - | - | - | - | - | 0.71 | - |
| STHS4 | - | - | - | - | - | 0.23 | 0.33 | - | - | - | - |
| STHS5 | - | - | - | - | - | - | 0.31 | - | - | - | - |
| STHS6. | - | - | - | - | - | - | - | - | - | 0.72 | - |
| STHS7. | - | - | - | - | - | - | - | - | - | 0.78 | - |
| STHS8 | - | - | - | - | - | - | 0.28 | - | - | - | - |
| STHS9. | - | - | - | - | - | - | - | - | - | 0.75 | - |
| STHS10 | - | - | - | - | - | 0.21 | 0.32 | - | - | - | - |
| TBS1 | - | 0.35 | 0.21 | - | - | - | - | - | - | - | - |
| TBS2 | - | 0.34 | - | - | - | - | - | - | - | - | - |
| TBS3 | - | 0.3 | - | - | - | - | - | - | 0.23 | - | - |
| TBS4 | - | 0.37 | - | - | - | - | 0.2 | - | 0.23 | - | - |
| TBS5 | - | 0.31 | - | - | - | - | 0.21 | - | - | - | - |
| TBS6 | - | 0.38 | - | - | - | - | - | - | - | - | - |
| TBS7 | - | 0.42 | - | - | - | - | - | - | 0.21 | - | - |
| TBS8 | - | 0.41 | - | - | - | - | - | - | 0.22 | - | - |
| PB.VAS | - | - | - | - | - | - | - | - | - | - | - |
| DS1 | - | - | - | - | - | - | 0.51 | - | - | - | - |
| DS2. | - | - | - | - | - | - | - | - | - | 0.47 | - |
| DS3 | - | - | - | - | - | - | 0.51 | - | - | - | - |
| DS4. | - | - | - | - | - | - | 0.22 | - | - | 0.46 | - |
| DS5 | - | - | - | - | - | - | 0.46 | - | - | - | - |
| DS6 | - | - | - | - | - | - | 0.45 | - | - | - | - |
| DS7 | - | - | - | - | - | - | 0.5 | - | - | - | - |
| DS8 | - | - | - | - | - | - | 0.44 | - | - | - | - |
| DS9. | - | - | - | - | - | - | - | - | 0.24 | 0.48 | - |
| DS10 | - | - | - | - | - | - | 0.49 | - | - | - | - |
| DS11 | - | - | - | - | - | - | 0.45 | - | - | - | - |
| DS12 | - | - | - | - | - | - | 0.51 | - | - | - | - |
| DS13 | - | - | - | - | - | - | 0.45 | - | - | - | - |
| DS14 | - | - | - | - | - | - | 0.45 | - | - | - | - |
| DS15 | - | - | - | - | - | - | 0.48 | - | - | - | - |
| DS16 | - | - | - | - | - | - | 0.44 | - | - | - | - |
| TRAP1 | - | - | - | - | - | 0.4 | - | - | - | - | - |
| TRAP2 | - | - | - | - | - | 0.41 | - | - | - | - | - |
| TRAP3 | - | - | 0.21 | - | - | 0.48 | - | - | - | - | - |
| TRAP4 | - | - | 0.25 | - | - | 0.44 | - | - | - | - | - |
| TRAP5 | - | - | - | - | - | 0.5 | - | - | - | - | - |
| TRAP6 | - | - | - | - | - | 0.45 | - | - | - | - | - |
| TRAP7 | - | - | 0.2 | - | - | 0.47 | - | - | - | - | - |
| TRAP8 | - | - | - | - | - | 0.53 | - | - | - | - | - |
| TRAP9 | - | - | - | - | - | 0.48 | - | - | - | - | - |
| TRAP10 | - | - | - | - | - | 0.53 | - | - | - | - | - |
| TRAP11 | - | - | - | - | - | 0.51 | - | - | - | - | - |
| TRAP12 | - | - | - | - | - | 0.51 | - | - | - | - | - |
| TRAP13 | - | - | - | - | - | 0.52 | - | - | - | - | - |
| TRAP14 | - | - | - | - | - | 0.43 | - | - | - | - | - |
| TRAP15 | - | - | - | - | - | 0.52 | - | - | - | - | - |
| TRAP16 | 0.21 | - | - | - | - | 0.48 | - | - | - | - | - |
| HUM1 | 0.64 | - | - | - | - | - | - | - | - | - | - |
| HUM2 | 0.72 | - | - | - | - | - | - | - | - | - | 0.21 |
| HUM3 | 0.75 | - | - | - | - | - | - | - | - | - | - |
| HUM4 | 0.71 | - | - | - | - | - | - | - | - | - | - |
| HUM5 | 0.78 | - | - | - | - | - | - | - | - | - | - |
| HUM6 | 0.8 | - | - | - | - | - | - | - | - | - | - |
| HUM7 | 0.82 | - | - | - | - | - | - | - | - | - | - |
| HUM8 | 0.81 | - | - | - | - | - | - | - | - | - | - |
| HUM9 | 0.82 | - | - | - | - | - | - | - | - | - | - |
| HUM10 | 0.83 | - | - | - | - | - | - | - | - | - | - |
| HUM11 | 0.88 | - | - | - | - | - | - | - | - | - | - |
| HUM12 | 0.83 | - | - | - | - | - | - | - | - | - | - |
| HUM13 | 0.83 | - | - | - | - | - | - | - | - | - | - |
| HUM14 | 0.89 | - | - | - | - | - | - | - | - | - | - |
| HUM15 | 0.9 | - | - | - | - | - | - | - | - | - | - |
| HUM16 | 0.8 | - | - | - | - | - | - | - | - | - | - |
| HUM17 | 0.92 | - | - | - | - | - | - | - | - | - | - |
| HUM18 | 0.84 | - | - | - | - | - | - | - | - | - | - |
| HUM19 | 0.82 | - | - | - | - | - | - | - | - | - | - |
| HUM20 | 0.87 | - | - | - | - | - | - | - | - | - | - |
| HUM21 | 0.88 | - | - | - | - | - | - | - | - | - | - |
| HUM22 | 0.85 | - | - | - | - | - | - | - | - | - | - |
| HUM23 | 0.79 | - | - | - | - | - | - | - | - | - | - |
| HUM24 | 0.86 | - | - | - | - | - | - | - | - | - | - |
| HUM25 | 0.83 | - | - | - | - | - | - | - | - | - | - |
| HUM26 | 0.77 | - | - | - | - | - | - | - | - | - | - |
| HUM27 | 0.76 | - | - | - | - | - | - | - | - | - | - |
| HUM28 | 0.75 | - | - | - | - | - | - | - | - | - | - |
| HUM29 | 0.82 | - | - | - | - | - | - | - | - | - | - |
| HUM30 | 0.73 | - | - | - | - | - | - | - | - | - | - |
| HUM31 | 0.7 | - | - | - | - | - | - | - | - | - | - |
| HUM32 | 0.67 | - | - | - | - | - | - | - | - | - | - |
| Pain1 | - | - | 0.77 | - | - | - | - | - | - | - | - |
| Pain2 | - | - | 0.77 | - | - | - | - | - | - | - | - |
| Pain3 | - | - | 0.81 | - | - | - | - | - | - | - | - |
| Pain4 | - | - | 0.76 | - | - | - | - | - | - | - | - |
| Pain5 | - | - | 0.78 | - | - | - | - | - | - | - | - |
| Pain6 | - | - | 0.6 | - | - | - | - | - | - | - | - |
| Pain7 | - | - | 0.74 | - | - | - | - | - | - | - | - |
| Pain8 | - | - | 0.63 | - | - | - | - | - | - | - | - |
| Pain9 | - | - | 0.72 | - | - | - | - | - | - | - | - |
| Pain10 | - | - | 0.69 | - | - | - | - | - | - | - | - |
| Pain11 | - | - | 0.67 | - | - | - | - | - | - | - | - |
| Pain12 | - | - | 0.71 | - | - | - | - | - | - | - | - |
| Pain13 | - | - | 0.69 | - | - | - | - | - | - | - | - |
| Anger1 | - | - | - | 0.65 | - | - | - | - | - | - | - |
| Anger2 | - | - | - | 0.63 | - | - | - | - | - | - | - |
| Anger3 | 0.23 | - | - | 0.36 | - | - | - | - | - | - | - |
| Anger4 | - | - | - | 0.64 | - | - | - | - | - | - | - |
| Anger5 | - | - | - | 0.73 | - | - | - | - | - | - | - |
| Anger6 | - | - | - | 0.54 | - | - | - | - | - | - | - |
| Anger7 | - | - | - | 0.56 | - | - | - | - | - | - | - |
| Anger8 | - | - | - | 0.57 | - | - | - | - | - | - | - |
| Anger9 | - | - | - | 0.57 | - | - | - | - | - | - | - |
| Anger10 | - | - | - | 0.53 | - | - | - | - | - | - | - |
| Anger11 | - | - | - | 0.49 | - | - | - | - | - | - | - |
| Anger12 | - | - | - | 0.56 | - | - | - | - | - | - | - |
| Anger13 | - | - | - | 0.59 | - | - | - | - | - | - | - |
| Anger14 | - | - | - | 0.61 | - | - | - | - | - | - | - |
| Anger15 | - | - | - | 0.68 | - | - | - | - | - | - | - |
| Anger16 | - | - | - | 0.7 | - | - | - | - | - | - | - |
| Anger17 | - | - | - | 0.74 | - | - | - | - | - | - | - |
| Anger18 | - | - | - | 0.69 | - | - | - | - | - | - | - |
| Anger19 | - | - | - | 0.56 | - | - | - | - | - | - | - |
| Anger20 | - | - | - | 0.5 | - | - | - | - | - | - | - |
| Anger21 | - | - | - | 0.68 | - | - | - | - | - | - | - |
| Anger22 | - | - | - | 0.61 | - | - | - | - | - | - | - |
| Agg1 | - | - | 0.21 | 0.22 | - | - | - | - | - | - | 0.26 |
| Agg2 | - | - | - | - | 0.29 | - | - | - | - | - | 0.35 |
| Agg3 | - | - | - | - | 0.28 | - | - | - | - | - | 0.41 |
| Agg4 | - | - | - | - | 0.29 | - | - | - | - | - | 0.36 |
| Impulse1 | - | - | - | - | 0.65 | - | - | - | - | - | - |
| Impulse2 | - | - | - | - | 0.65 | - | - | - | - | - | - |
| Impulse3 | - | - | - | - | 0.74 | - | - | - | - | - | - |
| Impulse4 | - | - | - | - | 0.8 | - | - | - | - | - | - |
| Impulse5 | - | - | - | - | 0.78 | - | - | - | - | - | - |
| Impulse6 | - | - | - | - | 0.72 | - | - | - | - | - | - |
| Impulse7 | - | - | - | - | 0.69 | - | - | - | - | - | - |
| Impulse8 | - | - | - | - | 0.67 | - | - | - | - | - | - |
| Impulse9 | - | - | - | - | 0.71 | - | - | - | - | - | - |
| Impulse10 | - | - | - | - | 0.69 | - | - | - | - | - | - |
| Impulse11. | - | - | - | 0.26 | - | - | - | - | 0.21 | 0.42 | - |
| Impulse12 | - | - | - | - | 0.7 | - | - | - | - | - | - |
| DT1 | - | - | - | - | - | - | - | 0.44 | - | - | - |
| DT2 | - | - | 0.22 | - | - | - | - | 0.43 | - | - | - |
| DT3 | - | - | - | - | - | - | - | 0.48 | - | - | - |
| DT4 | - | - | - | - | - | - | - | 0.45 | - | - | - |
| DT5 | - | - | - | - | - | - | - | 0.53 | - | - | - |
| DT6. | - | - | - | - | - | - | - | - | 0.28 | 0.25 | - |
| DT7 | - | - | - | - | - | - | - | 0.6 | - | - | - |
| DT8 | - | - | - | - | - | - | - | 0.76 | - | - | - |
| DT9 | - | - | - | - | - | - | - | 0.51 | - | - | - |
| DT10 | - | - | - | - | - | - | - | 0.57 | - | - | - |
| DT11 | - | - | - | - | - | - | - | 0.51 | - | - | - |
| DT12 | - | - | - | - | - | - | - | 0.5 | - | - | - |
| DT13 | - | - | - | - | - | - | - | 0.71 | - | - | - |
| DT14 | - | - | - | - | - | - | - | 0.77 | - | - | - |
| DT15 | - | - | - | - | - | - | - | 0.51 | - | - | - |
| BH.N1 | - | - | - | - | - | - | 0.32 | - | - | - | - |
| BH.N2 | - | - | - | - | - | - | 0.28 | - | - | - | - |
| BH.N3 | - | - | - | - | - | - | 0.3 | - | - | - | - |
| BH.P1. | - | - | - | - | - | - | - | - | - | 0.67 | - |
| BH.P2. | - | - | - | - | - | - | - | - | - | 0.68 | - |
| BH.P3. | - | - | - | - | - | - | - | - | - | 0.66 | - |

Note: Item abbreviations followed by . denote reverse coded items; - = loadings < .20; INQ = Interpersonal Needs Questionnaire, UCLA = UCLA Loneliness, STHS = State-Trait Hopelessness Scale, TBS = Thwarted Belongingness Scale, PB.VAS = Perceived Burdensomeness Visual Analog Scale Item (Bryan, 2019), DS = Defeat Scale, TRAP = Entrapment Scale, HUM = Humiliation, Pain = Psychological Pain, Agg = Aggression, Impulse = Negative Urgency, DT = Distress Tolerance, BH.N = Brief Hopelessness Negative, BH.P = Brief Hopelessness Positive.

**Table S5**. Factor Loading Patterns for 10-dimensional EIFA in Reduced Item Pool

|  | F1 | F2 | F3 | F4 | F5 | F6 | F7 | F8 | F9 | F10 |
| --- | --- | --- | --- | --- | --- | --- | --- | --- | --- | --- |
| INQ1 | - | 0.23 | 0.24 | - | - | - | 0.24 | - | - | 0.24 |
| INQ2 | - | 0.25 | - | - | - | - | 0.2 | - | - | 0.25 |
| INQ3 | - | 0.22 | 0.25 | - | - | - | 0.21 | - | - | 0.23 |
| INQ4 | - | 0.22 | 0.23 | - | - | - | 0.24 | - | - | 0.32 |
| INQ5 | 0.2 | 0.24 | - | - | - | - | 0.22 | - | - | 0.28 |
| INQ6 | - | 0.21 | 0.29 | - | - | - | 0.23 | - | - | - |
| INQ7. | - | - | - | - | - | - | - | 0.73 | - | - |
| INQ8. | - | - | - | - | - | - | - | 0.67 | - | - |
| INQ9. | - | - | - | - | - | - | - | 0.8 | - | - |
| INQ10. | - | - | - | - | - | - | - | 0.75 | - | - |
| INQ11 | - | 0.4 | - | - | - | - | - | - | - | - |
| INQ12 | - | 0.36 | - | - | - | - | - | - | - | - |
| INQ13. | - | - | - | - | - | - | - | 0.79 | - | - |
| INQ14. | - | - | - | - | - | - | - | 0.75 | - | - |
| INQ15. | - | - | - | - | - | - | - | 0.75 | - | - |
| UCLA1 | - | 0.58 | - | - | - | - | - | - | - | - |
| UCLA2 | - | 0.74 | - | - | - | - | - | - | - | - |
| UCLA3 | - | 0.54 | - | - | - | - | - | - | - | - |
| UCLA4 | - | 0.81 | - | - | - | - | - | - | - | - |
| UCLA5 | - | 0.68 | - | - | - | - | - | - | - | - |
| UCLA6 | - | 0.59 | - | - | - | - | - | - | - | - |
| UCLA7 | - | 0.73 | - | - | - | - | - | - | - | - |
| UCLA8 | - | 0.75 | - | - | - | - | - | - | - | - |
| UCLA9 | - | 0.68 | - | - | - | - | - | - | - | - |
| UCLA10 | - | 0.68 | - | - | - | - | - | - | - | - |
| UCLA11 | - | 0.69 | - | - | - | - | - | - | - | - |
| UCLA12 | - | 0.64 | - | - | - | - | - | - | - | - |
| UCLA13 | - | 0.61 | - | - | - | - | - | - | - | - |
| UCLA14 | - | 0.6 | - | - | - | - | - | - | - | - |
| UCLA15 | - | 0.71 | - | - | - | - | - | - | - | - |
| UCLA16 | - | 0.72 | - | - | - | - | - | - | - | - |
| UCLA17 | - | 0.52 | - | - | - | - | - | - | - | - |
| UCLA18 | - | 0.62 | - | - | - | - | - | - | - | - |
| UCLA19 | - | 0.61 | - | - | - | - | - | - | - | - |
| UCLA20 | - | 0.59 | - | - | - | - | - | - | - | - |
| STHS1 | - | - | - | - | - | - | 0.31 | - | - | - |
| STHS2 | - | 0.23 | - | - | - | - | 0.32 | - | - | - |
| STHS3. | - | - | - | - | - | - | - | - | 0.67 | - |
| STHS4 | - | - | - | - | - | - | 0.36 | - | 0.21 | - |
| STHS5 | - | - | - | - | - | - | 0.32 | - | - | - |
| STHS6. | - | - | - | - | - | - | - | - | 0.69 | - |
| STHS7. | - | - | - | - | - | - | - | - | 0.75 | - |
| STHS8 | - | - | - | - | - | - | 0.3 | - | - | - |
| STHS9. | - | - | - | - | - | - | - | - | 0.72 | - |
| STHS10 | - | 0.21 | - | - | - | - | 0.34 | - | - | - |
| TBS1 | - | 0.39 | 0.23 | - | - | - | - | - | - | - |
| TBS2 | - | 0.39 | - | - | - | - | - | - | - | - |
| TBS3 | - | 0.35 | - | - | - | - | - | - | - | - |
| TBS4 | - | 0.43 | - | - | - | - | - | - | - | - |
| TBS5 | - | 0.36 | - | - | - | - | - | - | - | - |
| TBS6 | - | 0.43 | - | - | - | - | - | - | - | - |
| TBS7 | - | 0.47 | - | - | - | - | - | - | - | - |
| TBS8 | - | 0.46 | - | - | - | - | - | - | - | - |
| PB.VAS | - | - | 0.21 | - | - | - | 0.2 | - | - | - |
| DS1 | - | - | - | - | - | - | 0.35 | - | - | - |
| DS2. | - | - | - | - | - | - | - | - | 0.52 | - |
| DS3 | - | - | - | - | - | - | 0.35 | - | 0.21 | - |
| DS4. | - | - | - | - | - | - | - | - | 0.51 | - |
| DS5 | - | - | - | - | - | - | 0.31 | - | - | - |
| DS6 | - | - | - | - | - | - | 0.34 | - | - | - |
| DS7 | - | - | - | - | - | - | 0.35 | - | 0.2 | - |
| DS8 | - | - | - | - | - | - | 0.29 | - | - | - |
| DS9. | - | - | - | - | - | - | - | 0.27 | 0.47 | - |
| DS10 | - | - | - | - | - | 0.22 | 0.33 | - | 0.21 | - |
| DS11 | - | - | - | - | - | - | 0.29 | - | - | - |
| DS12 | - | - | - | - | - | - | 0.34 | - | 0.22 | - |
| DS13 | - | - | - | - | - | - | 0.3 | - | - | - |
| DS14 | - | - | - | - | - | - | 0.31 | - | - | - |
| DS15 | - | - | - | - | - | - | 0.39 | - | - | - |
| DS16 | - | - | - | - | - | 0.22 | 0.32 | - | - | - |
| TRAP1 | - | - | 0.24 | - | - | - | 0.28 | - | - | - |
| TRAP2 | - | - | - | - | 0.22 | - | 0.35 | - | - | - |
| TRAP3 | - | - | 0.28 | - | - | - | 0.32 | - | - | - |
| TRAP4 | - | - | 0.32 | - | - | - | 0.29 | - | - | - |
| TRAP5 | - | - | 0.26 | - | - | - | 0.36 | - | - | - |
| TRAP6 | - | - | 0.25 | - | - | - | 0.36 | - | - | - |
| TRAP7 | - | - | 0.27 | - | - | - | 0.33 | - | - | - |
| TRAP8 | - | - | 0.26 | - | - | - | 0.37 | - | - | - |
| TRAP9 | - | - | - | - | 0.29 | - | 0.24 | - | - | - |
| TRAP10 | - | - | - | - | - | - | 0.35 | - | - | - |
| TRAP11 | - | - | - | - | - | - | 0.41 | - | - | - |
| TRAP12 | - | - | - | 0.2 | - | - | 0.39 | - | - | - |
| TRAP13 | - | - | - | - | - | - | 0.43 | - | - | - |
| TRAP14 | 0.22 | - | - | - | - | - | 0.28 | - | - | - |
| TRAP15 | - | - | - | - | - | - | 0.33 | 0.21 | - | - |
| TRAP16 | 0.25 | - | - | - | - | - | 0.29 | - | - | - |
| HUM1 | 0.65 | - | - | - | - | - | - | - | - | - |
| HUM2 | 0.74 | - | - | - | - | - | - | - | - | 0.21 |
| HUM3 | 0.77 | - | - | - | - | - | - | - | - | - |
| HUM4 | 0.72 | - | - | - | - | - | - | - | - | - |
| HUM5 | 0.8 | - | - | - | - | - | - | - | - | - |
| HUM6 | 0.82 | - | - | - | - | - | - | - | - | - |
| HUM7 | 0.84 | - | - | - | - | - | - | - | - | - |
| HUM8 | 0.83 | - | - | - | - | - | - | - | - | - |
| HUM9 | 0.84 | - | - | - | - | - | - | - | - | - |
| HUM10 | 0.85 | - | - | - | - | - | - | - | - | - |
| HUM11 | 0.9 | - | - | - | - | - | - | - | - | - |
| HUM12 | 0.86 | - | - | - | - | - | - | - | - | - |
| HUM13 | 0.85 | - | - | - | - | - | - | - | - | - |
| HUM14 | 0.91 | - | - | - | - | - | - | - | - | - |
| HUM15 | 0.92 | - | - | - | - | - | - | - | - | - |
| HUM16 | 0.82 | - | - | - | - | - | - | - | - | - |
| HUM17 | 0.94 | - | - | - | - | - | - | - | - | - |
| HUM18 | 0.87 | - | - | - | - | - | - | - | - | - |
| HUM19 | 0.84 | - | - | - | - | - | - | - | - | - |
| HUM20 | 0.89 | - | - | - | - | - | - | - | - | - |
| HUM21 | 0.9 | - | - | - | - | - | - | - | - | - |
| HUM22 | 0.87 | - | - | - | - | - | - | - | - | - |
| HUM23 | 0.81 | - | - | - | - | - | - | - | - | - |
| HUM24 | 0.88 | - | - | - | - | - | - | - | - | - |
| HUM25 | 0.85 | - | - | - | - | - | - | - | - | - |
| HUM26 | 0.79 | - | - | - | - | - | - | - | - | - |
| HUM27 | 0.78 | - | - | - | - | - | - | - | - | - |
| HUM28 | 0.77 | - | - | - | - | - | - | - | - | - |
| HUM29 | 0.84 | - | - | - | - | - | - | - | - | - |
| HUM30 | 0.75 | - | - | - | - | - | - | - | - | - |
| HUM31 | 0.72 | - | - | - | - | - | - | - | - | - |
| HUM32 | 0.69 | - | - | - | - | - | - | - | - | - |
| Pain1 | - | - | 0.83 | - | - | - | - | - | - | - |
| Pain2 | - | - | 0.83 | - | - | - | - | - | - | - |
| Pain3 | - | - | 0.86 | - | - | - | - | - | - | - |
| Pain4 | - | - | 0.81 | - | - | - | - | - | - | - |
| Pain5 | - | - | 0.83 | - | - | - | - | - | - | - |
| Pain6 | - | - | 0.64 | - | - | - | - | - | - | - |
| Pain7 | - | - | 0.78 | - | - | - | - | - | - | - |
| Pain8 | - | - | 0.68 | - | - | - | - | - | - | - |
| Pain9 | - | - | 0.77 | - | - | - | - | - | - | - |
| Pain10 | - | - | 0.74 | - | - | - | - | - | - | - |
| Pain11 | - | - | 0.72 | - | - | - | - | - | - | - |
| Pain12 | - | - | 0.75 | - | - | - | - | - | - | - |
| Pain13 | - | - | 0.74 | - | - | - | - | - | - | - |
| Anger1 | - | - | - | 0.7 | - | - | - | - | - | - |
| Anger2 | - | - | - | 0.67 | - | - | - | - | - | - |
| Anger3 | 0.24 | - | - | 0.39 | - | - | - | - | - | - |
| Anger4 | - | - | - | 0.69 | - | - | - | - | - | - |
| Anger5 | - | - | - | 0.79 | - | - | - | - | - | - |
| Anger6 | - | - | - | 0.57 | - | - | - | - | - | - |
| Anger7 | - | - | - | 0.6 | - | - | - | - | - | - |
| Anger8 | - | - | - | 0.61 | - | - | - | - | - | - |
| Anger9 | - | - | - | 0.61 | - | - | - | - | - | - |
| Anger10 | - | - | - | 0.57 | - | - | - | - | - | - |
| Anger11 | - | - | - | 0.53 | - | - | - | - | - | - |
| Anger12 | - | - | - | 0.61 | - | - | - | - | - | - |
| Anger13 | - | - | - | 0.63 | - | - | - | - | - | - |
| Anger14 | - | - | - | 0.65 | - | - | - | - | - | - |
| Anger15 | - | - | - | 0.73 | - | - | - | - | - | - |
| Anger16 | - | - | - | 0.75 | - | - | - | - | - | - |
| Anger17 | - | - | - | 0.8 | - | - | - | - | - | - |
| Anger18 | - | - | - | 0.74 | - | - | - | - | - | - |
| Anger19 | - | - | - | 0.6 | - | - | - | - | - | - |
| Anger20 | - | - | - | 0.53 | - | - | - | - | - | - |
| Anger21 | - | - | - | 0.73 | - | - | - | - | - | - |
| Anger22 | - | - | - | 0.66 | - | - | - | - | - | - |
| Agg1 | - | - | 0.23 | 0.24 | - | - | - | - | - | 0.25 |
| Agg2 | - | - | - | - | 0.29 | - | - | - | - | 0.35 |
| Agg3 | - | - | - | - | 0.27 | - | - | - | - | 0.41 |
| Agg4 | - | - | - | - | 0.29 | - | - | - | - | 0.36 |
| Impulse1 | - | - | - | - | 0.64 | - | - | - | - | - |
| Impulse2 | - | - | - | - | 0.64 | - | - | - | - | - |
| Impulse3 | - | - | - | - | 0.74 | - | - | - | - | - |
| Impulse4 | - | - | - | - | 0.8 | - | - | - | - | - |
| Impulse5 | - | - | - | - | 0.79 | - | - | - | - | - |
| Impulse6 | - | - | - | - | 0.73 | - | - | - | - | - |
| Impulse7 | - | - | - | - | 0.69 | - | - | - | - | - |
| Impulse8 | - | - | - | - | 0.66 | - | - | - | - | - |
| Impulse9 | - | - | - | - | 0.72 | - | - | - | - | - |
| Impulse10 | - | - | - | - | 0.68 | - | - | - | - | - |
| Impulse11. | - | - | - | 0.27 | - | - | - | 0.24 | 0.39 | - |
| Impulse12 | - | - | - | - | 0.7 | - | - | - | - | - |
| DT1 | - | - | - | - | - | 0.46 | - | - | - | - |
| DT2 | - | - | 0.23 | - | - | 0.45 | - | - | - | - |
| DT3 | - | - | - | - | - | 0.5 | - | - | - | - |
| DT4 | - | - | - | - | - | 0.47 | - | - | - | - |
| DT5 | - | - | - | - | - | 0.55 | - | - | - | - |
| DT6. | - | - | - | - | - | - | - | 0.31 | 0.21 | - |
| DT7 | - | - | - | - | - | 0.62 | - | - | - | - |
| DT8 | - | - | - | - | - | 0.81 | - | - | - | - |
| DT9 | - | - | - | - | - | 0.54 | - | - | - | - |
| DT10 | - | - | - | - | - | 0.59 | - | - | - | - |
| DT11 | - | - | - | - | - | 0.54 | - | - | - | - |
| DT12 | - | - | - | - | - | 0.52 | - | - | - | - |
| DT13 | - | - | - | - | - | 0.74 | - | - | - | - |
| DT14 | - | - | - | - | - | 0.81 | - | - | - | - |
| DT15 | - | - | - | - | - | 0.53 | - | - | - | - |
| BH.N1 | - | - | - | - | - | - | 0.27 | - | - | - |
| BH.N2 | - | 0.23 | - | - | - | - | 0.27 | - | 0.23 | - |
| BH.N3 | - | - | - | - | - | - | 0.3 | - | - | - |
| BH.P1. | - | - | - | - | - | - | - | - | 0.66 | - |
| BH.P2. | - | - | - | - | - | - | - | - | 0.68 | - |
| BH.P3. | - | - | - | - | - | - | - | - | 0.64 | - |

Note: Item abbreviations followed by . denote reverse coded items; - = loadings < .20; INQ = Interpersonal Needs Questionnaire, UCLA = UCLA Loneliness, STHS = State-Trait Hopelessness Scale, TBS = Thwarted Belongingness Scale, PB.VAS = Perceived Burdensomeness Visual Analog Scale Item (Bryan, 2019), DS = Defeat Scale, TRAP = Entrapment Scale, HUM = Humiliation, Pain = Psychological Pain, Agg = Aggression, Impulse = Negative Urgency, DT = Distress Tolerance, BH.N = Brief Hopelessness Negative, BH.P = Brief Hopelessness Positive.

**Table S6.** EMA Compliance Rates over Study Weeks.

| ID | Week 1 | Week 2 | Week 3 | Overall | ID | Week 1 | Week 2 | Week 3 | Overall |
| --- | --- | --- | --- | --- | --- | --- | --- | --- | --- |
| 21 | 50.00% | 45.24% | 45.24% | 46.83% | 37 | 42.86% | 54.76% | 50.00% | 49.21% |
| 22 | 52.38% | 9.52% | 0% | 20.63% | 38 | 73.81% | 69.05% | 73.81% | 71.43% |
| 23 | 59.52% | 57.14%. | 59.52% | 58.73% | 40 | 83.33% | 83.33% | 76.19% | 80.95% |
| 25 | 76.19% | 69.05% | 52.38% | 65.87% | 41 | 59.52% | 80.95% | 73.81% | 71.43% |
| 26 | 73.81% | 61.90% | 54.76% | 63.49% | 42 | 64.29% | 11.90% | 16.67% | 30.95% |
| 27 | 78.57% | 73.81% | 69.05% | 73.81% | 43 | 57.14% | 33.33% | 57.14% | 49.21% |
| 28 | 85.71% | 83.33% | 78.57% | 82.54% | 44 | 50.00% | 4.76% | 7.14% | 20.63% |
| 29 | 59.52% | 54.76% | 54.76% | 56.35% | 45 | 78.57% | 85.71% | 59.52% | 74.60% |
| 30 | 69.05% | 64.29% | 45.24% | 59.52% | 46 | 69.05% | 66.67% | 47.62% | 61.11% |
| 31 | 83.33% | 78.57% | 83.33% | 81.75% | 47 | 45.24% | 33.33% | 38.10% | 38.89% |
| 32 | 78.57% | 66.67% | 64.29% | 69.84% | 48 | 73.81% | 50.00% | 30.95% | 51.59% |
| 33 | 88.10% | 80.95% | 73.81% | 80.95% | 49 | 50.00% | 28.57% | 28.57% | 35.71% |
| 34 | 80.95% | 76.19% | 80.95% | 79.37% | 50 | 66.67% | 54.76% | 7.14% | 42.86% |
| 35 | 38.10% | 21.43% | 16.67% | 25.40% | 51 | 47.62% | 47.62% | 9.52% | 34.92% |
| 36 | 83.33% | 40.48% | 0% | 41.27% | - | - | - | - | - |

**Table S7.** Survey Content Similarity.

| ID | Jaccard Index^1^ | Overlap Coefficient^1^ | Surveys Complete | Mean Survey Length |
| --- | --- | --- | --- | --- |
| 21 | 0.356 | 0.496 | 64 | 48.5 |
| 22 | 0.274 | 0.383 | 80 | 46.9 |
| 23 | 0.511 | 0.635 | 26 | 47.3 |
| 25 | 0.688 | 0.279 | 104 | 16.6 |
| 26 | 0.722 | 0.313 | 98 | 18.6 |
| 27 | 0.350 | 0.483 | 83 | 47.5 |
| 28 | 0.367 | 0.370 | 116 | 34.9 |
| 29 | 0.476 | 0.612 | 62 | 50.0 |
| 30 | 0.211 | 0.327 | 90 | 48.9 |
| 31 | 0.658 | 0.764 | 78 | 48.9 |
| 32 | 0.239 | 0.365 | 104 | 49.5 |
| 33 | 0.349 | 0.490 | 54 | 48.7 |
| 34 | 0.382 | 0.524 | 103 | 49.9 |
| 35 | 0.233 | 0.362 | 76 | 49.8 |
| 36 | 0.316 | 0.456 | 35 | 48.6 |
| 37 | 0.276 | 0.292 | 96 | 36.9 |
| 38 | 0.450 | 0.594 | 91 | 50.0 |
| 40 | 0.216 | 0.325 | 96 | 47.3 |
| 41 | 0.301 | 0.353 | 39 | 38.5 |
| 42 | 0.198 | 0.313 | 105 | 49.0 |
| 43 | 0.314 | 0.284 | 63 | 30.6 |
| 44 | 0.376 | 0.493 | 29 | 46.0 |
| 45 | 0.337 | 0.276 | 101 | 2834 |
| 46 | 0.768 | 0.861 | 81 | 50.0 |
| 47 | 0.271 | 0.388 | 67 | 46.5 |
| 48 | 0.363 | 0.370 | 46 | 34.8 |
| 49 | 0.238 | 0.355 | 49 | 49.4 |
| 50 | 0.469 | 0.249 | 44 | 20.2 |
| 51 | 0.325 | 0.273 | 54 | 29.6 |
| Median | 0.349 | 0.370 | 78 | 47.27* |
| IQR | 0.176 | 0.179 | 42 | 14.12* |
| Mean | 0.380 | 0.424 | 73.59 | 41.79* |
| SD | 0.156 | 0.152 | 26.02 | 10.57* |
| ^1^mean of all pairwise values IQR = interquartile range; SD = standard deviation; *note that survey length was bimodal so interpreting central tendency measures directly may be misleading | | | | |
